# Comparative metagenomic study unveils new insights on bacterial communities in two pine-feeding *Ips* beetles (Coleoptera: Curculionidae: Scolytinae)

**DOI:** 10.3389/fmicb.2024.1400894

**Published:** 2024-10-09

**Authors:** Arunabha Khara, Amrita Chakraborty, Roman Modlinger, Jiří Synek, Amit Roy

**Affiliations:** Faculty of Forestry and Wood Sciences, Czech University of Life Sciences Prague, Prague, Czechia

**Keywords:** *Ips acuminatus*, *Ips sexdentatus*, core bacteriome, microhabitat, holobiont, amplicon sequence variances (ASVs)

## Abstract

**Background:**

Climate change has recently boosted the severity and frequency of pine bark beetle attacks. The bacterial community associated with these beetles acts as “hidden players,” enhancing their ability to infest and thrive on defense-rich pine trees. There is limited understanding of the environmental acquisition of these hidden players and their life stage-specific association with different pine-feeding bark beetles. There is inadequate knowledge on novel bacterial introduction to pine trees after the beetle infestation. Hence, we conducted the first comparative bacterial metabarcoding study revealing the bacterial communities in the pine trees before and after beetle feeding and in different life stages of two dominant pine-feeding bark beetles, namely *Ips sexdentatus* and *Ips acuminatus*. We also evaluated the bacterial association between wild and lab-bred beetles to measure the deviation due to inhabiting a controlled environment.

**Results:**

Significant differences in bacterial amplicon sequence variance (ASVs) abundance existed among different life stages within and between the pine beetles. However, *Pseudomonas, Serratia, Pseudoxanthomonas, Taibaiella,* and *Acinetobacter* served as core bacteria. Interestingly, *I. sexdentatus* larvae correspond to significantly higher bacterial diversity and community richness and evenness compared to other developmental stages, while *I. acuminatus* adults displayed higher bacterial richness with no significant variation in the diversity and evenness between the life stages. Both wild and lab-bred *I. sexdentatus* beetles showed a prevalence of the bacterial family *Pseudomonadaceae.* In addition, wild *I. sexdentatus* showed dominance of *Yersiniaceae,* whereas *Erwiniaceae* was abundant in lab-bred beetles. Alternatively, *Acidobacteriaceae*, *Corynebacteriaceae*, and *Microbacteriaceae* were highly abundant bacterial families in lab-bred, whereas *Chitinophagaceae* and *Microbacteriaceae* were highly abundant in wild *I. accuminatus.* We validated the relative abundances of selected bacterial taxa estimated by metagenomic sequencing with quantitative PCR.

**Conclusion:**

Our study sheds new insights into bacterial associations in pine beetles under the influence of various drivers such as environment, host, and life stages. We documented that lab-breeding considerably influences beetle bacterial community assembly. Furthermore, beetle feeding alters bacteriome at the microhabitat level. Nevertheless, our study revisited pine-feeding bark beetle symbiosis under the influence of different drivers and revealed intriguing insight into bacterial community assembly, facilitating future functional studies.

## Introduction

Bark beetles (*Coleoptera: Curculionidae: Scolytinae*) are economically important forest pests that cause large-scale forest damage across Europe (Biedermann et al., 2019; Singh et al., 2024). The outbreaks of pine bark beetles such as *Ips sexdentatus* (Börner, 1776) and *Ips acuminatus* (Gyllenhal, 1827) heavily impact forestry economics, affecting the forest-dependent sector and international wood markets (Montagné-Huck and Brunette, 2018). These outbreaks reduce the forest tree lifespan, decrease carbon uptake, and alter microclimatic situations in the forest, along with recreational and aesthetic values (Abdullah et al., 2018; Dobor et al., 2018). According to recent reports, particularly in the Czech Republic, the severity of pinewood damage by bark beetles increased by over 80,000 m^3^ in 2019 from about 10,000 m^3^ in 2009 (Liška et al., 2021). Moreover, climate changes such as increased air temperature, altered precipitation patterns, and increased frequency of drought and heat events have critically stimulated the outbreaks of bark beetles by altering the tree defence physiology (Raffa et al., 2008; Marini et al., 2017). Precisely, drought affects host tree fitness and can stimulate bark-beetle populations to overcome the epidemic threshold and cause an outbreak (McNichol et al., 2022). For instance, high spring temperature stimulates *I. acuminatus* to infest weakened and vigorous trees (Chinellato et al., 2014). Subsequently, the increasing infestations by *I. acuminatus* have listed the species among the most-aggressive bark beetle species within Europe (Faccoli et al., 2012; Colombari et al., 2013; Plewa and Mokrzycki, 2017; Liška et al., 2021). Climate change has also stimulated the importance of *I. sexdentatus* as a forest pest with considerable dispersal competency (Pineau et al., 2017b).

Although *I. sexdentatus* and *I. acuminatus* share several ecological characteristics, including host, *I. sexdentatus* infests the lower part of the bole, whereas *I. acuminatus* attacks the higher bark canopy (top branches) (Davydenko et al., 2017). Nonetheless, both *Ips* species must overcome the robust pine defence system to thrive. The robust pine defence system has physical and chemical barriers against biotic stress, including bark beetles (Mumm and Hilker, 2006). Physical barriers include tissues having lignin and suberin polymers that offer protection against degradation, penetration and ingestion, while the chemical barriers involve phenolic compounds and terpenoids (monoterpenes, diterpenes, and sesquiterpenes) with entomotoxic competency. A high concentration of monoterpenes shows ovicidal, repellent, adulticidal, and larvicidal effects against bark beetles (Krokene, 2015). Therefore, the defence barrier in healthy pine trees is a formidable challenge for bark beetles. Alternatively, gut microbial assemblage in bark beetles might promote the competency of bark beetles to exhaust conifer defence (Chakraborty et al., 2020a; Chakraborty et al., 2020b). Bacterial genera such as *Pseudomonas*, *Serratia*, and *Rahnella* associated with *Dendroctonus valens* (LeConte, 1860) might play an important role in metabolising monoterpenes (Boone et al., 2013; Xu et al., 2016). Similarly, *Erwinia typography* isolated from *Ips typographus* (Linnaeus, 1758) is suggested to tolerate a high concentration of myrcene-a plant defensive monoterpene (Skrodenytė-Arbačiauskienė et al., 2012). Furthermore, mountain pine beetle (*Dendroctonus ponderosae* Hopkins) has been reported to have bacterial mutualists containing genes (i.e., dit genes-diterpene degrading genes) that could be associated with terpene degradation (Adams et al., 2013).

Apart from overcoming the tree defence, a significant challenge for bark beetles is nutrient acquisition, as they primarily thrive on phloem tissues with limited nutrition and complex carbohydrates. Subsequently, the gut microbiome comes to the rescue, helping the beetles to digest and metabolise complex polysaccharides (i.e., lignin from conifers) (Hu et al., 2014) and nutrient acquirement (Morales-Jiménez et al., 2009; Morales-Jiménez et al., 2013). Beetle-associated microbiome extends their symbiosis with the host by producing pheromones, thus facilitating chemical communications (Xu et al., 2015). Furthermore, beetle-bacterial mutualism also plays an important role in defence against pathogens (González-Dominici et al., 2021). However, such microbial contribution to bark beetle survival is limited to only a few beetle genera, such as the red turpentine and mountain pine beetle (Adams et al., 2013; Cheng et al., 2018). Nevertheless, our understanding of bark beetles as holobionts is restricted. Few studies have evaluated microbial acquisition and succession in bark beetles (Ibarra-Juarez et al., 2020; Veselská et al., 2023; Baños-Quintana et al., 2024). In addition, limited studies in pine bark beetles have focused on understanding the effect of environment and metamorphosis that can cause comprehensive changes in the beetle microbiome, resulting in distinct bacterial communities. Moreover, microbial community assembly at the level of bark beetle microhabitat is still lacking. There is limited information on the influence of the host plant microbiome in shaping the beetle bacterial community, particularly in terms of *Ips* bark beetles (Kolasa et al., 2019; Silver et al., 2021; Chakraborty et al., 2023; Pirttilä et al., 2023; Baños-Quintana et al., 2024). No literature highlights the impact of beetle feeding on the pine-associated microbiome. Subsequently, it is also crucial to assess the microbial assembly in wild and lab-bred beetles to evaluate the influence of the bark beetle breeding facility.

We hypothesised that metamorphosis, microhabitat, and lab-breeding might shape the bacterial community structure in the *Ips* pine beetles similar to other insects (Chen et al., 2018; González-Serrano et al., 2020; Tian et al., 2022; Chakraborty et al., 2023; Veselská et al., 2023; Baños-Quintana et al., 2024). Hence, we conducted the first comparative metabarcoding study on two pine-feeding beetles (*I. sexdentatus*, *I. acuminatus*) to evaluate our hypothesis and respond to the following fundamental questions regarding bark beetle symbiosis: (1) What are the bacterial communities associated with the two pine-feeding *Ips* beetles across life-stages? (2) What influence does the host microbiome have in shaping the bacterial community assemblage or vice versa? (3) How does lab-breeding impact pine beetle bacterial community assemblage?

## Materials and methods

### Bark beetle collection and breeding for life-stage specific sample collection

Adult bark beetles (Coleoptera: Curculionidae: Scolytinae) were collected from infested logs (dbh ~20 cm) obtained from different beetle colonies in the Rouchovany forest area (49.0704° N, 16.1076° E) in the Czech Republic in 2020. The two bark beetle species (*I.sexdentatus* and *I.acuminatus*) were identified based on the work of Nunberg (Nunberg, 1981) and Pfeffer (Pfeffer, 1955, 1995). The infested logs were brought to the debarking room directly from the forest, where the adult beetles were collected using surface disinfected tweezers and bred to F2 generation in laboratory conditions (temp around 25°C in the day and 19°C at night, RH 60%) on fresh pine logs collected from the same forest area. Over 50 samples from each life stage (larva, pupa, adult) were collected from multiple infested logs in 50 mL plastic conical tubes, snap-frozen with liquid nitrogen, and kept at −80°C for DNA extraction. The gallery wood was collected from the same F2 generation infested logs using surface sterilised blades and kept in plastic tubes with RNAlater solution at −80°C for future use. All the beetle and wood samples were collected at least 10 cm below the edges of the logs to minimise contamination (Table 1). Uninfected, fresh woods with no beetle infestation were taken as control wood (unfed fresh phloem) and were kept in plastic conical tubes with RNAlater solution at −80°C.

**Table 1:** Sample description.

Sample Code	Sample details	Collection	Replicates
Ctrl.W	Uninfested pine wood control for lab breeding	Under lab environment	4
Fed.W	*Ips sexdentatus* infested gallery wood from F2 generation	Under lab environment	4
ISX.Larvae	*Ips sexdentatus* larvae from F2 generation	Lab bred	5
ISX.Pupae	*Ips sexdentatus* pupae from F2 generation	Lab bred	5
ISX.Adult	*Ips sexdentatus* adults from F2 generation	Lab bred	5
ISX.WL.Adult	*Ips sexdentatus* wild adults	Collected from forest	5
ISX.Ctrl.W	Uninfested wood control for *Ips sexdentatus*	Collected from forest	4
ISX.Fed.W	*Ips sexdentatus* infested gallery wood	Collected from forest	4
IAC.Larvae	*Ips acuminatus* larvae from F2 generation	Lab bred	5
IAC.Pupae	*Ips acuminatus* pupae from F2 generation	Lab bred	5
IAC.Adult	*Ips acuminatus* adults from F2 generation	Lab bred	5
IAC.WL.Adult	*Ips acuminatus* wild adults	Collected from forest	5
IAC.Ctrl.W	Uninfested wood control for *Ips accuminatus*	Collected from forest	4
IAC.Fed.W	*Ips acuminatus* infested gallery or fed wood	Collected from forest	4

### Wild beetle collection

Similarly, the infested logs, collected from the same Rouchovany forest area in 2021, were directly brought to the debarking room, where adult *I. sexdentatus* and *I. acuminatus* wild beetles were collected using surface sterilised tweezers in 50 mL plastic conical tubes, snap-frozen with liquid nitrogen and kept at −80°C for DNA extraction. The gallery wood samples were collected from the same infested logs in RNAlater solution and stored at −80°C. Similarly, unfed wood samples (fresh phloem tissue) were collected from logs from the same locality without any beetle infestation. Beetle bacteriome variability of individual colonies was not explored in the current study due to the mixing of beetle samples from different colonies. However, such a sampling method can reduce the random variability from the heterologous sampling material. The sample details are provided in Table 1.

### DNA extraction

Developmental stage-specific lab-bred and wild-collected samples were randomly selected and disinfected by rinsing the beetles twice with 70% ethanol and subsequently washing them with sterile water. Any sample with apparent infection was discarded. Due to size differences, the sampling amount per replicate for both species differed. One individual per replicate was used for *I. sexdentatus*, whereas, for *I. acuminatus,* four larvae/replicate, two pupae/replicate, and five adults/replicate were used. Total DNA from whole beetles was extracted using the MACHEREY-NAGEL NucleoSpin Soil DNA kit with modifications in the manufacturer’s protocol. Similarly, wood microbial DNA was extracted (~120 mg wood/replicate) using QIAGEN DNeasy Plant Mini Kit following the manufacturer’s protocol with modifications. The samples were homogenised under liquid nitrogen, and the lysis was performed for 1 min. The extracted DNA quantity and quality were accessed using a Qubit 2.0 Fluorometer (Thermo Scientific) and 1% agarose gel electrophoresis. High-quality samples (five biological replicates from each life stage for both species and 4 biological replicates for each wood sample) and two negative extraction controls were selected for 16S rRNA gene metabarcoding at Novogene Company, China.

### 16S amplicon sequencing

16S amplicon sequencing was executed at Novogene, China, using a pre-optimised protocol. Precisely, 1 ng/μl template DNA, bacterial 16S rRNA gene primers (341F-806R) (Klindworth et al., 2013) containing unique barcodes, and Phusion High-Fidelity PCR Master Mix (New England Biolabs) were used to set up PCR reactions. Subsequently, a PCR reaction mixture without template DNA was used as a negative control. PCR products were visualised in 2% agarose gel electrophoresis. The samples with amplification between 450 and 480 bp were selected and mixed in equidensity ratios for gel purification using a QIAGEN Gel extraction kit. NEBNext Ultra DNA Library Pre-Kit for Illumina was used to create sequencing libraries followed by index code ligation. Sequencing libraries were quantitively and qualitatively analysed using Qubit 2.0 Fluorometer (Thermo Fisher Scientific) and Agilent Bioanalyser 2,100 system. An Illumina Novaseq 6,000 platform obtained 250 bp paired-end reads from the sequenced libraries.

### Bacteriome data analysis

#### Data processing and species annotation

The bioinformatic data analysis was performed using a standardised pipeline in QIIME2 (version 2022.2) (Bolyen et al., 2019) as described in our earlier studies (Gupta et al., 2023). The barcodes and primer sequences were removed, and the sample sequences were merged using FLASH (V1.2.11)^1^ to generate raw Illumina pair-end reads (Magoč and Salzberg, 2011) and then checked for high-quality reads using fastp software. VSEARCH software (Rognes et al., 2016) was used to identify and remove chimeric sequences. The chloroplast and mitochondrial sequences were discarded to facilitate downstream bioinformatic analyses. The amplicon sequence variant (ASV) (Li et al., 2020) abundance table was obtained using the DADA2 module (Callahan et al., 2016). The ASV abundance table or the feature table is a matrix of samples, and the feature (ASV) abundance represents the number of times each feature/ASV was observed in each sample. Sequences that have an abundance lesser than five were discarded. Furthermore, classify-sklearn algorithm, a pre-trained Naive Bayes classifier was used for species annotation with the bacterial SILVA database (version 138.1) (Quast et al., 2012; Bokulich et al., 2018) to obtain individual ASVs with respective species annotation in QIIME2 (version 2022.2) (Bolyen et al., 2019). The core consortium of the bacterial communities in the samples was defined as ASVs present in ≥60% of each beetle sample group to avoid the occurrence of any transient species.

#### Alpha diversity

Alpha diversity indices like bacterial community richness (Chao1), evenness (Pielou) (Magurran, 1988) and bacterial diversity (Shannon) (Magurran, 1988) were used to analyse bacterial community structure. Kruskal-Wallis-pairwise-group test was performed to test the significance level within the samples. In addition, QIIME2 (version 2022.2) was used to estimate Good’s coverage (sequence depth) (Chao et al., 1988) and observed species from all stage-specific and wood samples that were represented by R software (Version 2.15.3; R Core Team, 2013, Vienna, Austria) (R Core Team, 2013).

#### Beta diversity

The bacterial diversity variation between different life stages and wood samples for both *Ips* species was estimated using the UniFrac distance metric (Lozupone et al., 2011) determined in QIIME2 (version 2022.2). Moreover, unweighted UniFrac distance measurement was used for non-metric multi-dimensional scaling (NMDS) analysis illustrated by R software (Oksanen, 2007). The functions ADONIS (analysis and partitioning sum of squares using dissimilarities) and ANOSIM (analysis of similarities) (Clarke, 1993; Anderson, 2001) were used to determine significant differences in life-stage specific and wood bacteriome in QIIME2 (version 2022.2). ADONIS is a non-parametric multivariate variance test analysis that utilises a distance metric (Lozupone et al., 2011) to determine significant differences in the bacterial community among the sample groups (Stat et al., 2013). However, ANOSIM analysis utilises the same distance metric to estimate if the variation among different sample groups is larger than within the sample group (Chapman and Underwood, 1999). Additionally, a permutational multivariate analysis of variance (PERMANOVA) (Anderson, 2001) using Bray Curtis distance was conducted to assess the significance of overall bacterial diversity across different life stages and to evaluate the impact of lab-breeding on the two pine beetles. Furthermore, a *t*-test was used to determine significantly abundant bacterial species (*p* < 0.05) in the sample groups (D’Argenio et al., 2014). Similarly, Metastats analysis using false discovery rate (FDR) and multiple hypothesis tests for sparsely-sampled features revealed that the intra-group differed significantly on abundant bacterial species (Paulson et al., 2011). LEfSe (linear discriminant analysis effect size) analysis was performed to obtain significant biomarkers that can help to distinguish two samples in an experimental condition (Segata et al., 2011). These biologically consistent, statistically significant biomarkers derived from LEfSe can disclose metagenomic attributes (taxa/metabolites/genes) to distinguish between the two samples.

### Quantitative PCR assay

The relative abundance of selected bacterial taxa was estimated using quantitative PCR assay and correlated with the metagenomic sequencing results. Six individuals for each life stage (larvae/pupae/adults) were pooled per replicate, and four biological replicates were prepared. However, due to the limited availability of *I. acuminatus* samples, only four replicates of adult beetles were prepared. For the same reason, life stage comparisons for *I. acuminatus* were not performed using qPCR. Tubulin beta-1 chain (*β-Tubulin*) was used as reference genes for *I. sexdentatus* (Sellamuthu et al., 2021), while ribosomal protein (*RPL*7) and the elongation factor (*EF*1a) genes were selected for *I. accuminatus* (unpublished data). Six primer pairs representing different bacterial taxa and one eubacterial primer revealing the total bacterial population were used for qPCR assay (Supplementary Table 5). The specific bacterial primers were selected from previously published studies, while *Psedoxanthomonas* and *Serratia* genus-specific primers were designed in-house based on 16S rRNA gene sequences available in NCBI. The in-house designed primers were validated by sequencing the amplified product of the genus-specific primers and confirming it by NCBI blast. In addition, we also checked the primer with other non-specific bacterial cultures. The qPCR was performed with 10 μL of reaction mixture containing 4 μL of gDNA (10 ng/μl), 5 μL SYBR^®^ Green PCR Master Mix (Applied Biosystems), 0.5 μL forward and reverse primer (10 μM). Amplification conditions included initial denaturation at 95°C for 5 min, followed by 40 cycles of 95°C for 15 s and 60°C for 30 s. The relative quantification (RQ) of the selected bacterial population was estimated using the delta–delta Ct method (2^−ΔΔCt^), where ΔCt was estimated as the difference between the threshold Ct values with specific bacterial primers and the housekeeping reference gene. In this study, the 2^−ΔΔCt^ method reveals the fold change of the bacterial abundance relative to the housekeeping genes. It is worth mentioning here that the relative abundance of a specific bacterial population compared to the total bacterial population often lacks consistency (Navidshad et al., 2012). Hence, the housekeeping genes with stable gene expression were considered to normalise the data and determine the relative bacterial abundance. In the qPCR analysis, the significant difference in relative bacterial abundance between different life stages of *I. sexdentatus* and *I. acuminatus* was estimated using the method described by Pekár and Brabec (2016). The best linear model was elected under Akaike’s information criterion (AIC), and goodness of fit and heterogeneity were ensured by plotting residuals of the model against fitted values. Then, the variable was tested using ANOVA, and the difference between the categorical variable levels was compared by treatment contrasts *t*-test (Crawley, 2012). All analyses were performed in the R 4.3.1 environment (R Core Team, 2013).

## Results

### Bacteriome structure

#### Sequencing statistics

The sequencing data from two pine bark beetle species, *I. sexdentatus* (ISX) and *I. acuminatus* (IAC), of different populations (lab-bred, wild collection) and life stages (larva, pupa, adult) along with host tissue (control wood, fed/gallery wood) generated 6,393,911 raw reads. A Phred Quality score > 30 was used for quality control. Therefore, a total of 5,812,983 clean reads were obtained (Control wood-374,493; Fed wood-374,084; *I. sexdentatus* larvae-463,927; *I. sexdentatus* pupae-440,213; *I. sexdentatus* adult-446,527; *I. acuminatus* larvae-463,230; *I. acuminatus* pupae-446,017; *I. acuminatus* adult-372,076; *I. sexdentatus* wild adult-475,613; *I. sexdentatus* Control wood-364,782; *I. sexdentatus* Fed wood-381,812; *I. acuminatus* wild adult-447,180; *I. acuminatus* Control wood - 387, 312; *I. acuminatus* Fed wood-375,717) (Supplementary Excel 1).

### Bacterial relative abundance at distinct taxonomic levels

**Figure 1:**
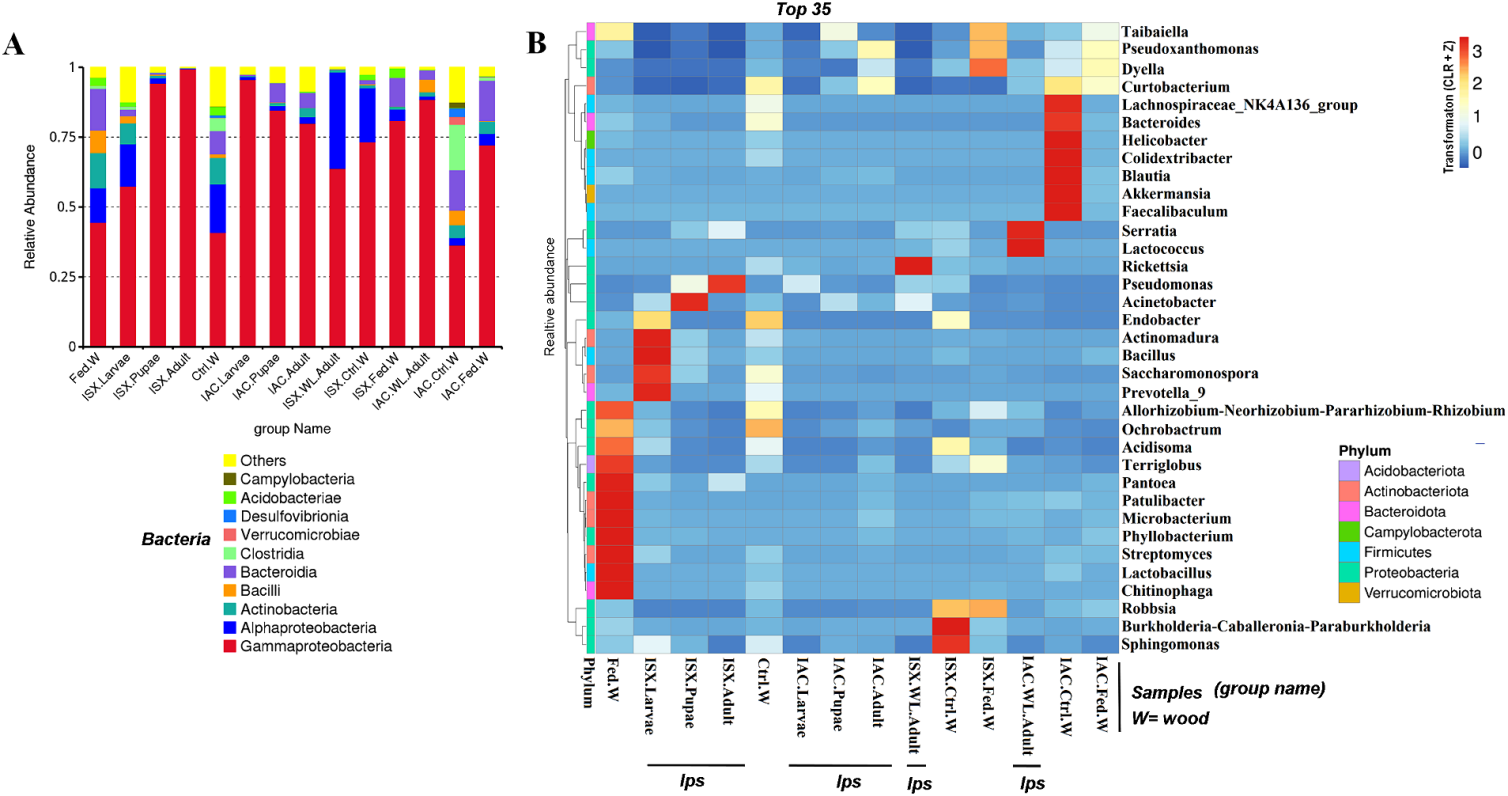
Bacterial diversity in lab-bred (life-stages), wild-type adult beetles and wood samples. **(A)** The bar plot represents the relative abundance of bacteriome at the class level (top 10). **(B)** Heatmap depicting the relative abundance of 35 dominant bacterial genera among lab-bred (life-stages), wild-type adult beetles and wood samples. The relative ASV abundance is represented by a colour gradient where the darker colour indicates higher abundance, whereas the lighter colour indicates low abundance for a specific bacterial genus (ISX-*Ips sexdentatus*, IAC-*Ips acuminatus*).

The bacterial sequences obtained from the two beetle species and wood samples generate 4,056 ASVs at a 100% similarity level (Supplementary Excel 2). The Good’s coverage indicator (>98%) and the rarefaction curve indicated sampling comprehensiveness that represented the bacterial communities associated with the beetle and wood samples (Table 2; Supplementary Figure 1). The estimation of the Goods coverage index (0.98) after filtering of sequence read (*n* > 5) suggests that the majority of the ASVs present in the samples were detected, and approximately only 2% of the ASVs were not covered during the sequencing. The predominant bacterial classes across all samples were Gammaproteobacteria, Alphaproteobacteria, Actinobacteria, Bacteroidia, Bacilli (Figure 1A). The relative abundance of Gammaproteobacteria was higher in *I. sexdentatus* pupae (0.94 ± 0.03) and adult (0.99 ± 0.002) samples compared to larvae (0.57 ± 0.15) and wild-collected adults (0.63 ± 0.20) samples (Supplementary Excel 2). Similarly, the relative abundance of Gammaproteobacteria was highest in *I. acuminatus* larvae (0.95 ± 0.004) compared to other stages (larvae-0.84 ± 0.06, pupae-0.79 ± 0.06) and wild-collected adults (0.88 ± 0.02). Furthermore, Gammaproteobacteria [*I. sexdentatus* Control wood (0.73 ± 0.03), *I. sexdentatus* Fed wood (0.80 ± 0.01), and *I. acuminatus* Control wood (0.36 ± 0.02) *I. acuminatus* Fed wood (0.72 ± 0.05)] was also the dominant class in different phloem wood samples (Supplementary Excel 2). The relative abundance of Alphaproteobacteria was documented as highest in *I. sexdentatus* wild-collected adults (0.34 ± 0.19) compared to all other samples (Supplementary Excel 2).

**Table 2:** Alpha diversity indices.

Samples	Good’s coverage (%)	Observed species	Chao1	Pielou	Shannon
Control wood (Ctrl.W)	98.1	224.25 ± 53.42	241 ± 64.22	0.82 ± 0.03	6.29 ± 0.43
Fed wood (Fed.W)	98.1	126.8 ± 18.94	166.3 ± 24.37	0.66 ± 0.04	4.57 ± 0.39
*Ips acuminatus* Larvae (IAC.Larvae)	98.5	80.6 ± 3.09	117.92 ± 13.59	0.60 ± 0.03	3.80 ± 0.15
*Ips acuminatus* Pupae (IAC.Pupae)	97.5	116.4 ± 30.83	178.64 ± 52.58	0.54 ± 0.04	3.65 ± 0.46
*Ips acuminatus* Adult (IAC.Adult)	96.4	157.4 ± 23.48	251.42 ± 51.88	0.51 ± 0.08	3.73 ± 0.66
*Ips acuminatus* Wild-collected Adult (IAC.WL.Adult)	98.3	89.4 ± 13.07	123.57 ± 19.99	0.48 ± 0.03	3.12 ± 0.28
*Ips acuminatus* Control wood (IAC.Ctrl.W)	99.5	144.25 ± 5.89	146.86 ± 5.42	0.84 ± 0.01	6.04 ± 0.11
*Ips acuminatus* Fed wood (IAC.Fed.W)	94.8	201 ± 96.89	336.40 ± 184.63	0.64 ± 0.03	4.71 ± 0.59
*Ips sexdentatus* Larvae (ISX.Larvae)	94.6	269.4 ± 81.03	396.9 ± 101.78	0.61 ± 0.14	4.99 ± 1.32
*Ips sexdentatus* Pupae (ISX.Pupae)	97.4	98.2 ± 24.94	181.37 ± 56.39	0.52 ± 0.04	3.30 ± 0.16
*Ips sexdentatus* Adult (ISX.Adult)	99.2	45.8 ± 4.54	63.32 ± 12.32	0.43 ± 0.07	2.34 ± 0.38
*Ips sexdentatus* Wild-collected Adult (ISX.WL.Adult)	98.2	78.2 ± 7.01	138.87 ± 12.60	0.43 ± 0.10	2.70 ± 0.62
*Ips sexdentatus* Control wood (ISX.Ctrl.W)	98.3	111 ± 14.65	161.33 ± 30.83	0.67 ± 0.03	4.48 ± 0.16
*Ips sexdentatus* Fed wood (ISX.Fed.W)	98.2	117 ± 8.22	152.62 ± 17.41	0.61 ± 0.00	4.20 ± 0.06

The data represented the mean value ±SE for five biological replicates across different life stages of two pine-feeding beetles and four biological replicates for the associated wood samples (SE-Standard Error).

### Life stage-specific bacteriome

#### Bacterial associations in *I. sexdentatus* (ISX)

**Figure 2:**
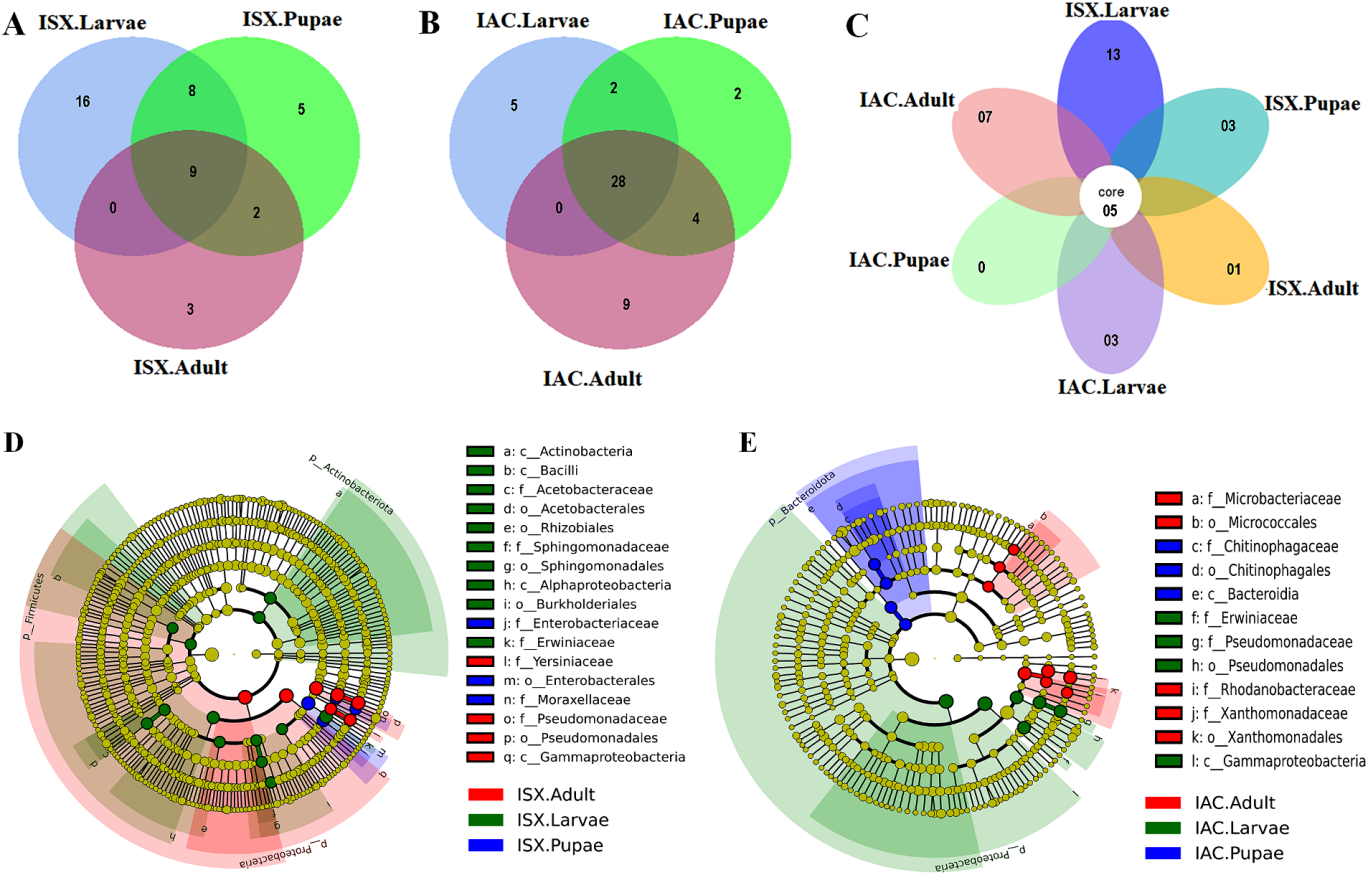
Core bacteriome. **(A)** Venn diagram illustrating bacterial ASV distribution in *I. sexdentatus* life stages (ISX.Larvae, ISX. Pupae, ISX.Adult). **(B)** Venn diagram depicting bacterial ASV distribution in *I. acuminatus* life stages (IAC.Larvae, IAC.Pupae, IAC.Adult). The shaded regions indicate the common areas between the sample groups. **(C)** Flower diagram representing the core ASVs across the life stages of two pine beetles. **(D)** Cladogram illustrating the results from LEfSe analysis revealing the biologically consistent, statistically significant bacterial biomarkers across different life stages of *I. sexdentatus* (ISX). **(E)** Cladogram representing significantly distinct bacterial biomarkers across *I. acuminatus* (IAC) life stages. Distinct taxonomic level (phylum to genus) is denoted in the circle from inward to outward. The different coloured nodes (red, green, and blue) represent bacterial species that play a significant role in different life stages across two different beetles (ISX and IAC), whereas yellowish-green circles represent non-significant bacterial species. Specific bacterial biomarkers are denoted by letters above the circles. The size of the nodes represents the relative abundance of the bacterial species at a particular taxon (ISX-*Ips sexdentatus*, IAC-*Ips acuminatus*).

Our study revealed that the ASV distribution of the different life stages of both pine beetles comprises a pool of diverse bacterial populations. *I. sexdentatus* larvae, pupae, and adult stages contained 16, 5, and 3 unique ASVs, respectively (Figure 2A). Moreover, nine common ASVs were present in all life stages of *I. sexdentatus*, constituting the core bacteriome. However, it is essential to mention that each ASV may not represent an individual species. The *I. sexdentatus* core consortium consisted of bacterial genera, including *Pseudomonas*, *Pseudoxanthomonas*, *Sphingomonas*, *Acinetobacter,* and members belonging to the bacterial family *Erwiniaceae* (Supplementary Excel 3). The heatmap revealed the high abundance of *Pseudomonas* in adults, whereas *Acinetobacter, Saccharomonospora,* and *Rickettsia* were dominant in pupae, larvae, and wild adults, respectively (Figure 1B). The alpha diversity analysis showed that *I. sexdentatus* adult beetles had substantially lower bacterial richness (Chao1 63.32 ± 12.32) than the other developmental stages (Chao1, larvae-396.9 ± 101.78, *p* < 0.01 and pupae-181.37 ± 56.39, *p* < 0.01) (Table 2; Supplementary Figure 2). However, *I. sexdentatus* larvae represented significantly higher bacterial diversity (Shannon-4.99 ± 1.32) compared to the adult (Shannon-2.34 ± 0.38, *p* < 0.01) (Table 2; Supplementary Figure 2). Similarly, the bacterial community evenness was significantly higher in *I. sexdentatus* larvae (Pielou-0.61 ± 0.14) compared to adults (Pielou-0.43 ± 0.07) (*p* < 0.05) (Table 2; Supplementary Figure 2). The overall bacterial diversity showed significant differences while comparing different developmental stages of *I. sexdentatus* (Permanova analysis, Pseudo F statistics = 5.399, *p* = 0.00003). Subsequently, Metastat analysis revealed the differential abundance of top bacterial genera between different developmental stages (Table 3). Among the bacterial genera present, *Pseudomonas* was the most dominant bacterial genus in all the three life stages of *I. sexdentatus* beetles, with differences in their relative abundance. LEfSe represented the key bacterial biomarkers in *I. sexdentatus* life stage-specific bacterial populations (Figure 2D; Supplementary Figure 3A; Supplementary Table 3). *I. sexdentatus* pupae documented the bacterial families, including *Enterobacteriaceae* and *Moraxellaceae,* as biomarkers (Figure 2D). In contrast, *Yersiniaceae* and *Pseudomonadaceae* were the biomarkers of *I. sexdentatus* adults. Similarly, *I. sexdentatus* larvae represented the class Alphaproteobacteria as a distinct biomarker (Supplementary Table 3).

**Table 3:** Metastat analysis representing the top 10 differently abundant bacterial genera across different life stages of *Ips* pine beetles.

Groups	Significantly different present bacterial genera (*p* < 0.05)
ISX.Larvae vs. ISX.Adult	*Pseudomonas, Serratia, Pantoea, Lactococcus*
ISX.Adult vs. ISX.Pupae	*Pseudomonas, Sphingomonas, Pantoea, Stenotrophomonas*
ISX.Larvae vs. ISX.Pupae	*Pseudomonas, Serratia, Lactococcus, Taibaiella, Acinetobacter*
IAC.Adult vs. IAC.Larvae	*Pseudomonas, Rickettsia, Curtobacterium, Arachidicoccus, Dyella*
IAC.Pupae vs. IAC.Adult	*Arachidicoccus, Dyella*
IAC.Larvae vs. IAC.Pupae	*Pseudomonas, Burkholderia-Caballeronia-Paraburkholderia*
IAC.Adult vs. ISX.Adult	*Pseudomonas, Serratia, Pantoea, Pseudoxanthomonas, Dyella, Curtobacterium, Arachidicoccus*
IAC.Pupae vs. ISX.Pupae	*Pseudomonas, Curtobacterium, Diaphorobacter, Serratia, Dyella, Burkholderia-Caballeronia-Paraburkholderia, Stenotrophomonas*
ISX.Larvae vs. IAC.Larvae	*Pseudomonas, Pseudoxanthomonas, Taibaiella, Dyella, Curtobacterium, Lactococcus*
ISX.Adult vs. ISX.WL.Adult	*Rickettsia, Pseudomonas, Pantoea, Rahnella*
IAC.Adult vs. IAC.WL.Adult	*Serratia, Lactococcus, Arachidicoccus, Allorhizobium-Neorhizobium-Pararhizobium-Rhizobium, Burkholderia-Caballeronia-Paraburkholderia, Carnimonas, Sphingobacterium*

The FDR test evaluates the significance of observed abundance differences among the samples (ISX.Larvae-*Ips sexdentatus* larvae; ISX.Pupae-*I. sexdentatus* pupae; ISX.Adult-I*. sexdendatus* adult; ISX.WL.Adult-I *sexdentatus* wild collected adults; IAC.Larvae-Ips *acuminatus* larvae; IAC.Pupae-*I. acuminatus* pupae; IAC.Adult-*I. acuminatus* adult; IAC.WL.Adult-I *acuminatus* wild collected adults).

### Bacterial associations in *I. acuminatus* (IAC)

The core bacteriome in *I. acuminatus* comprised 28 ASVs that were categorised into 10 families including *Erwiniaceae*, *Pseudomonadaceae, Xanthomonadaceae,* and *Rhodanobacteraceae* being the most dominant bacterial families (Figure 2B; Supplementary Excel 4). Comparing the developmental stages, *Pseudomonas* showed a high abundance in *I. acuminatus* larvae, while *Taibaiella, Sphingomonas,* and *Curtobacterium* were dominant in the pupal stage (Figure 1B). Furthermore, the alpha diversity indices revealed higher bacterial richness in *I. acuminatus* adults (Chao1, 251.42 ± 51.88) compared to larvae (Chao1, 117.92 ± 13.59) (*p* < 0.01) (Table 2; Supplementary Figure 2). However, no stage-specific differences in bacterial diversity and evenness were observed in *I. acuminatus* beetles (Table 2). Similar to *I. sexdentatus*, significant differences in the bacterial diversity were observed between *I. acuminatus* life stages (Permanova analysis Pseudo F statistics = 5.789, *p* = 0.00008). Metastat analysis revealed a significantly high abundance of *Pseudomonas* in *I. acuminatus* larvae, while *Arachidicoccus, Dyella* and *Burkholderia-Caballeronia-Paraburkholderia* were prevalent in adults and pupae (Table 3). Furthermore, LEfSe analysis corresponds to the bacterial biomarkers in *I. acuminatus* life stages (Figure 2E; Supplementary Figure 3B; Supplementary Table 3). *I. acuminatus* pupal biomarkers were categorised into class Bacteroidia (Figure 2E; Supplementary Table 3), while members from the phyla Proteobacteria (class Gammaproteobacteria, family *Erwiniaceae, Pseudomonadaceae*) were represented as the biomarkers in *I. acuminatus* larvae. Similarly, *I. acuminatus* adults documented family-Rhodanobactericeae, *Xanthomonadeceae* and *Microbactericeae* as predominant biomarkers (Supplementary Table 3).

### Comparing bacteriome of two pine-feeding Ips beetles

**Figure 3:**
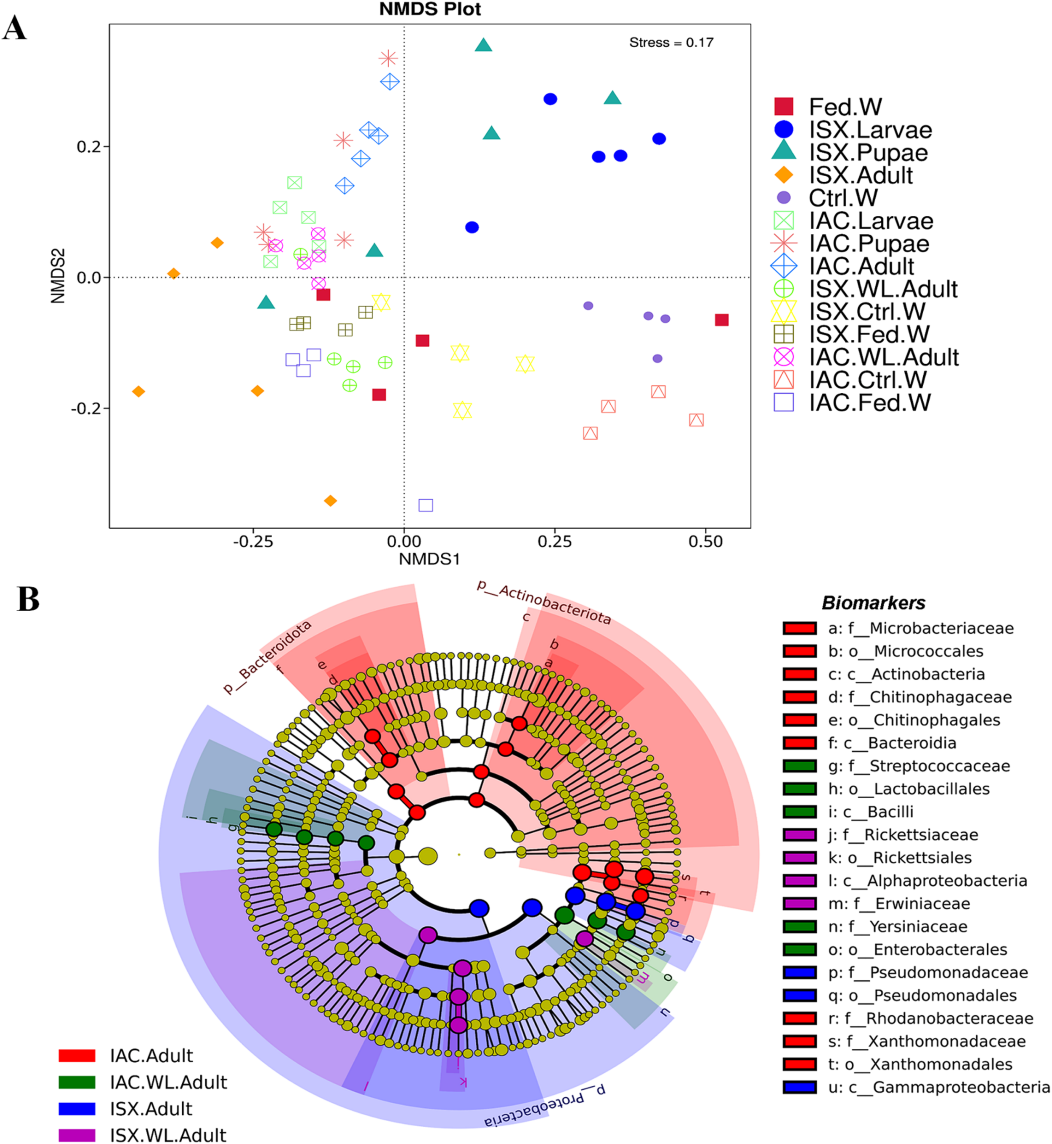
Impact of lab breeding on bacterial association. **(A)** Non-metric Multi-Dimensional Scaling (NMDS) based on unweighted UniFrac distance matrix represents bacterial diversity variation in different life stages of two beetles along with the wood samples. **(B)** Cladogram representing significant bacterial biomarkers among *I. sexdentatus, I. acuminatus* wild type and lab-bred beetles (ISX-*Ips sexdentatus*, IAC-*Ips acuminatus*). Distinct taxonomic level (phylum to genus) is denoted in the circle from inward to outward. The different coloured nodes (red, green, blue and purple) represent bacterial species that play a significant role in wild-collected adults and lab-reared adults in two different beetles (ISX and IAC), whereas yellowish-green circles represent non-significant bacterial species. Specific bacterial biomarkers are denoted by letters above the circles. The size of the nodes represents the relative abundance of the bacterial species at a particular taxon (ISX-*Ips sexdentatus*, IAC-*Ips acuminatus*).

Comparing the two beetle species, *I. acuminatus* and *I. sexdentatus,* 5 ASVs were shared across the developmental stages in both species (Figure 2C) that were assigned to 3 families including *Xanthomonadaceae*, *Erwiniaceae,* and *Sphingomonadaceae* (Supplementary Excel 5). Furthermore, the adult and larval stages of both species (*I. sexdentatus* and *I. acuminatus*) showed a significant difference (*p* < 0.01) in bacterial richness. For instance, in *I. sexdentatus* beetles, the larvae have higher bacterial richness, but in *I. acuminatus,* it was adult beetles. Although there was not much variation in bacterial diversity and evenness within the pupal and the larval stages between the two beetle species (*I. acuminatus* and *I. sexdentatus*), *I. acuminatus* adult showed significantly higher bacterial diversity (Shannon-3.73 ± 0.66) compared to *I. sexdentatus* adult (Shannon-2.34 ± 0.38, *p* < 0.05) (Table 2; Supplementary Figure 2). Additionally, NMDS using unweighted UniFrac distances revealed the differences between *I. sexdentatus* and *I. acuminatus* bacterial communitie*s* by hierarchically clustering different life stages and wood samples (Figure 3A). The larval and adult beetle-associated bacteria in *I. acuminatus* and *I. sexdentatus* had significant differences within and among them. Similarly, ADONIS and ANOSIM analysis revealed significant differences between the developmental stages of the two pine-feeding beetles (Supplementary Tables 1, 2).

### Impact of beetle lab breeding on bacterial assemblage

#### *I. sexdentatus:* wild vs. lab-bred

According to our study, the lab-bred adult population (F2 generation) and the wild beetle population have considerable differences in ASV composition. The *I. sexdentatus* wild and lab-bred adults had 7, 1 unique ASVs, and 22 shared ASVs (Supplementary Excel 6). The core bacterial consortium accounted for 6 families, including *Xanthomonadaceae, Erwiniaceae, Yersiniaceae,* and *Pseudomonadaceae* as the dominant families (Supplementary Figure 5; Supplementary Excel 6), while *Pseudomonas* was the highly abundant genera. Alpha diversity comparisons revealed that in *Ips sexdentatus* samples, the lab-bred adult beetles (Chao1-63.32 ± 12.32) had lower bacterial richness than wild adults (Chao1-138.87 ± 12.60, *p* < 0.05), while no significant variation was observed in the bacterial diversity and community evenness (Table 2; Supplementary Figure 2). However, lab-breeding showed a significant influence on the bacterial beta diversity in *I. sexdentatus* adults (Permanova analysis, Pseudo F statistics = 4.816, *p* = 0.00794) Metastat analysis revealed significant differences in bacterial abundance between the lab-bred and wild-collected beetles (Table 3). Additionally, the lab-bred and wild-collected beetles possess distinct bacterial markers (Figure 3B; Supplementary Figure 3C). For instance, LEfSe analysis revealed the members of the class Gammaproteobacteria, family-Pseudomonadaceae as the biomarkers of lab-bred adults, while class Alphaproteobacteria, family-Erwiniaceae, and *Rickettsiaceae* was represented as the biomarker of wild-collected adults (Figure 3B; Supplementary Table 4).

#### *I. acuminatus:* wild vs. lab-bred

The lab-bred and wild-collected *I. acuminatus* adult beetles comprised 30 common bacterial ASVs belonging to *Xanthomonadaceae, Erwiniaceae, Pseudomonadaceae*, *Rhodanobacteraceae*, *Chitinophagaceae*, and *Microbacteriaceae* (Supplementary Excel 6). The heatmap indicated that *Curtobacterium, Dyella,* and *Pseudoxanthomonas* dominated lab-bred adults, while *Taibaiella, Lactococcus,* and *Serratia* were prevalent in wild *I. acuminatus* beetles (Figure 1B). In contrast to *I. sexdentatus*, the alpha diversity analysis revealed that lab-bred *I. acuminatus* adult (Chao1 251.42 ± 51.88) had higher bacterial richness compared to wild adults (123.57 ± 19.99) (*p* < 0.05) (Table 2; Supplementary Figure 2). However, no significant differences in bacterial diversity and evenness were observed (Table 2; Supplementary Figure 2). Beta diversity analysis revealed significant differences between the bacterial communities in the lab-bred and wild-collected *I. acuminatus* adults (Permanova analysis, Pseudo F statistics = 6.389, *p* = 0.00794). LEfSe analysis represented biomarkers in *I. acuminatus* adult belonging to different phyla-Actinobacterioda (class-Actinobacteria; order-Micrococcales; family-Microbacteriaceae), Bacteroidota (class-Bacteroidia; order-Chitinophagales; family-Chitinophagaceae), Proteobacteria (family-*Rhodanobactericeae*, *Xanthomonadeceae*) (Figure 3B; Supplementary Figure 3C; Supplementary Table 4). *I. acuminatus* wild adults contained biomarkers belonging to two phyla-Proteobacteria (order-Enterobacterales; family-Yersiniaceae) and Firmicutes (class Bacilli; order-Lactobacillales; family-Streptococcaceae). Nevertheless, the wild adult beetles for both *Ips* species were clustered separately from lab-bred adults in the NMDS plot, indicating laboratory breeding impact on beetle bacteriome (Figure 3A). The ADONIS and ANOSIM results also demonstrate significant differences between the bacterial communities associated with wild and lab-bred beetles (Supplementary Tables 1, 2).

### Host contribution in shaping beetle bacteriome

**Figure 4:**
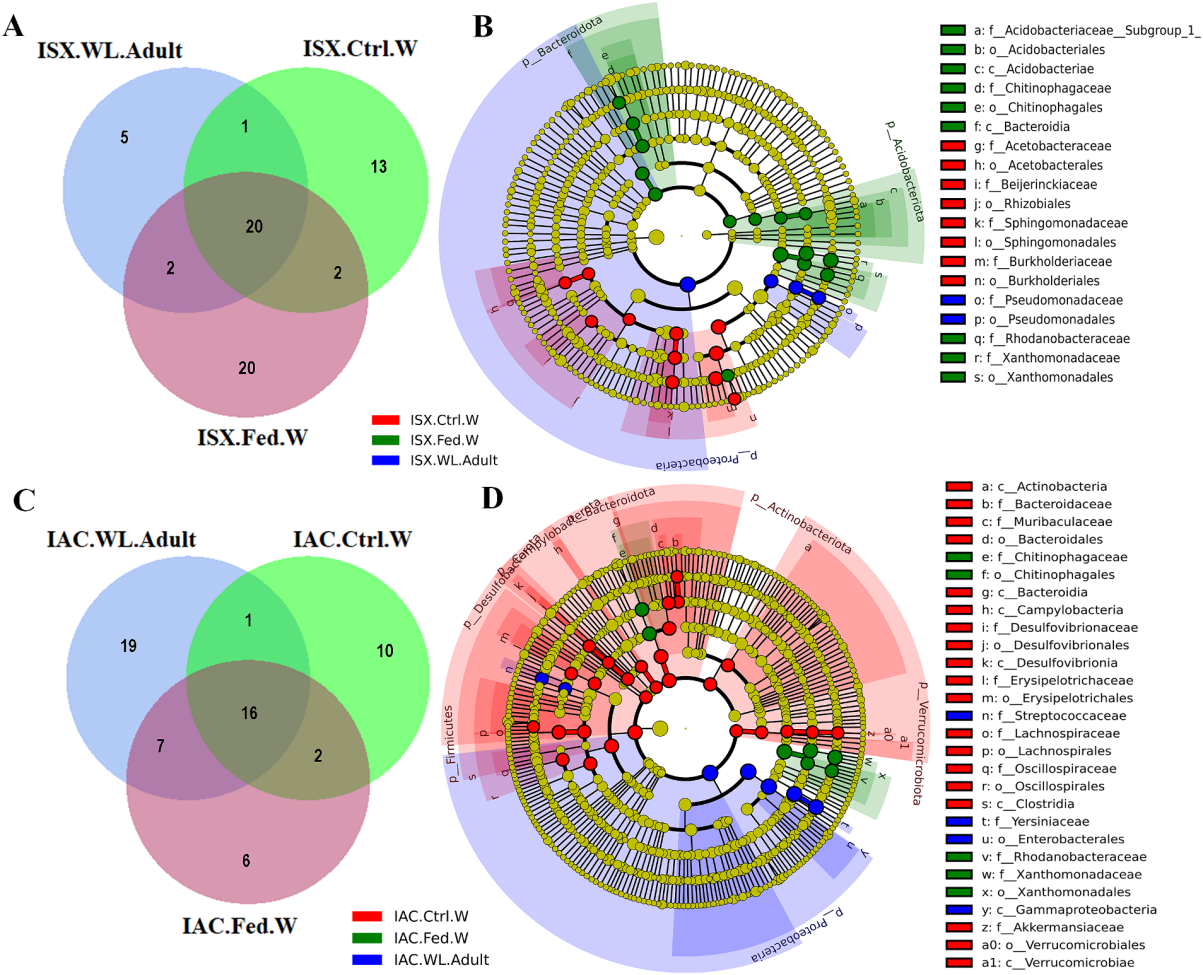
Host contribution in shaping beetle bacteriome. **(A)** Venn diagram showing the bacterial ASVs contribution of wood bacteriome in shaping *I. sexdentatus* bacteriome. **(B)** Cladogram representing significant bacterial biomarkers in *I. sexdentatus* wild-adult beetle and different wood types (control wood and fed wood). **(C)** Venn diagram showing the bacterial ASVs contribution of wood bacteriome in shaping *I. acuminatus* bacteriome. **(D)** Cladogram representing significant bacterial biomarkers among *I. acuminatus* wild adult beetle and different wood types (control wood and fed wood) (ISX-*Ips sexdentatus*, IAC-*Ips acuminatus*).

Comparing the *I. sexdentatus* wild beetles with their control and fed woods revealed a consortium of 20 shared ASVs (Figure 4A; Supplementary Excel 7). The shared bacteriome documented a high abundance of bacterial families *Pseudomonadaceae, Yersiniaceae, and Erwiniaceae,* while *Pseudomonas* is the dominant genus (Supplementary Excel 7). The unfed pine wood (*I. sexdentatus* Control wood) from the forest documented a high abundance of bacterial genera-Burkholderia_Caballeronia_Paraburkholderia whereas *Pseudoxanthomonas, Robbsia* were dominant in gallery wood of the wild *I. sexdentatus* (Figure 1B).

Similarly, considering the *I. acuminatus* wild beetles with their control and fed wood documented 16 shared ASVs belonging to bacterial families *Xanthomonadaceae*, *Erwiniaceae*, *Rhodanobacteraceae*, *Chitinophagaceae*, *Burkholderiaceae*, *Acetobacteraceae* (Figure 4C; Supplementary Excel 8). The heatmap revealed the prevalence of *Pseudoxanthomonas, Taibaiella, Dyella* in gallery wood of the wild *I. acuminatus*, which in turn had a lower abundance in unfed control pine wood (*I. acuminatus* Control wood), indicating an alteration in pine wood bacterial assemblage after beetle feeding (Figure 1B).

Several biomarkers were found while comparing the wild adult and wood bacterial communities. For instance, Proteobacteria was a significant biomarker with high abundance in the wild adult population (*I. sexdentatus* and *I. acuminatus* wild adult) (Figures 4B,D; Supplementary Figures 4A,B; Supplementary Table 3). Other biomarkers such as bacterial orders-Burkholderiales (family-Burkholderiaceae) and Pseudomonadales showed low abundance in *I. sexdentatus* fed wood, while an increase in the relative abundance of bacterial biomarkers belonging to bacterial family *Chitinophagaceae*, *Rhodanobacteraceae*, *Xanthomonadaceae* were observed compared to the control wood (Supplementary Table 3). Subsequently, biomarkers in *I. sexdentatus* wild adults were categorised into the family-Pseudomonadaceae, order-Pseudomonadales, and the biomarkers for *I. acuminatus* wild adults were classified into the family-Streptococcaceae, *Yersiniaceae*, order-Enterobacterales, class-Gammaproteobacteria. Concomitantly, in *I. acuminatus* control wood samples Bacterodiota, Dessulfobacteriota, Verrucomicrobiota were the significant biomarkers predominated by families like *Bacterodiaceae*, *Desulfovibrionaceae, Akkermansiaceae*. However, in the fed wood samples from two beetle species (*I. sexdentatus* and *I. acuminatus* fed wood), Proteobacteria, Acidobacteriota, and Bacterodiota were the predominant phyla. Similarly, there were differences at other taxa levels. LefSe analysis revealed that *I. sexdentatus* fed-wood biomarkers belonged to families such as *Acidobacteriaceae* _(Subgroup_1), *Chitinophagaceae*, *Rhodanobacteraceae*, and *Xanthomonadaceae* (Figure 4B; Supplementary Table 3). Similarly, *Chitinophagaceae*, *Rhodanobacteraceae*, and *Xanthomonadaceae* were the prevalent biomarkers in *I. acuminatus* fed wood (Figure 4D; Supplementary Table 3). Investigating the functional relevance of these biomarkers in different sample groups will be intriguing.

Alpha diversity analysis revealed higher bacterial diversity in control wood samples compared to fed wood. However, no significant difference was observed between wood samples (Table 2; Supplementary Figure 2). In contrast, the *I. acuminatus* fed/gallery wood samples collected from the forest demonstrated significantly different lower bacterial community evenness (*I. acuminatus* Fed wood-0.64 ± 0.03) than its respective control wood samples (*I. acuminatus* Control wood 0.84 ± 0.01) (*p* < 0.05) (Table 2; Supplementary Figure 2). Such findings indicate the enrichment of bacterial species in the feeding gallery after beetle feeding.

**Figure 5:**
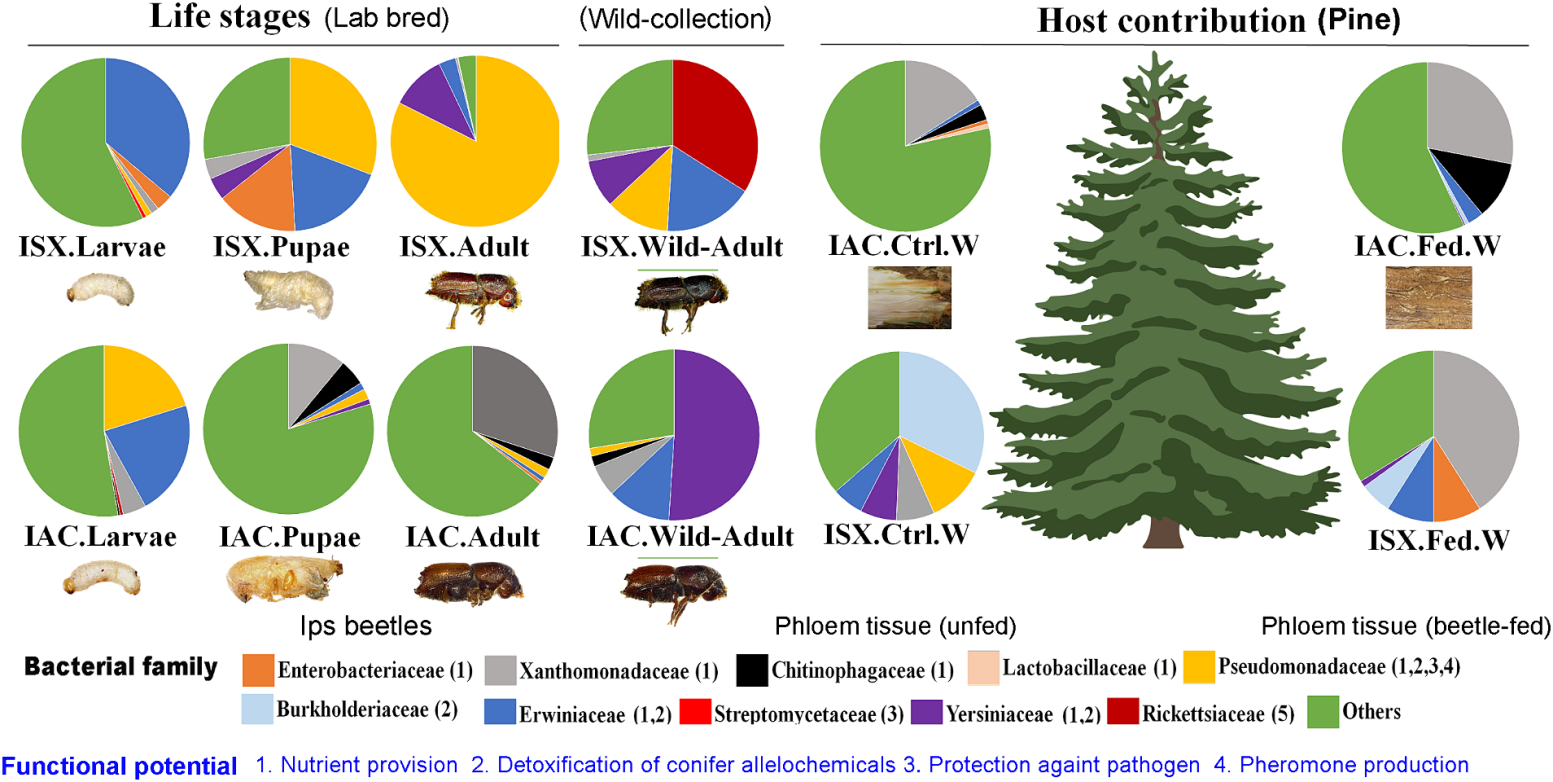
Schematic diagram containing the top five bacterial families at each developmental stage of two pine beetles (ISX-*Ips sexdentatus*, IAC-*I. acuminatus*) and their respective wood samples (control wood, gallery wood).

Consequently, the NMDS plot represented *I. acuminatus* wild adults, fed wood, and control wood into distinct clusters (Figure 3A). However, no such clustering was observed in the case of wild-collected *I. sexdentatus* samples. In contrast, comparing lab-bred and wild-collected wood samples (control wood and fed wood) for the *I. sexdentatus* revealed a distinct bacterial population, suggesting the influence of environment and beetle feeding as drivers in shaping the host microbiome (Figure 3A; Supplementary Excel 9; Supplementary Tables 1, 2). A schematic diagram containing the top five bacterial families across the developmental stages of two pine beetles and their respective wood and their putative role in the beetle holobiont is illustrated as a summary figure (Figure 5).

### Relative bacterial abundance using quantitative PCR assay

#### *I. sexdentatus* life stages

**Figure 6:**
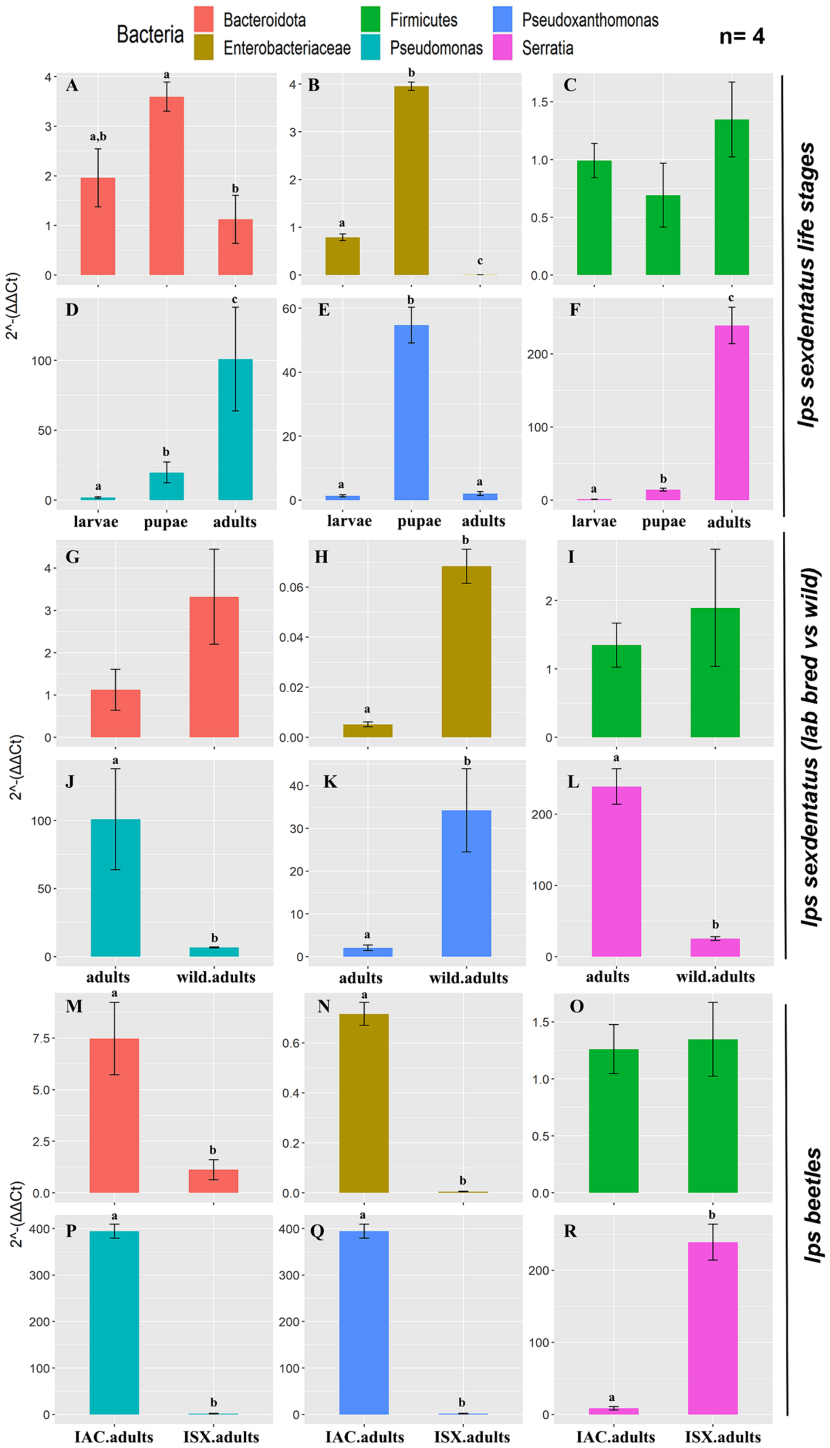
Quantitative PCR assay representing the relative abundance of selected bacterial taxa present in the two pine-feeding beetles. The 2^−ΔΔCt^ revealed the fold change of the bacterial abundance relative to the stable reference genes (ISX-*β-Tubulin*; IAC-EF1a, *RPL7*). The data indicates the mean fold change of bacterial abundance within the beetles (*n* = 4). The data was tested using ANOVA, and the difference between the categorical variable levels was compared using the treatment contrasts *t*-test. Different letters indicate the statistical significance, *p* < 0.01. **(A–F)** *I. sexdentatus* life stage comparisons; **(G–L)** *I. sexdentatus* wild vs. lab-bred adults; **(M–R)** *I. sexdentatus* vs. *I. accuminatus* lab-bred adults. The statistical analysis results were presented in Supplementary Excel 10.

The qPCR assay revealed a relatively high abundance of total bacterial population (eubacterial primers) in *I. sexdentatus* adults compared to all other life stages (Supplementary Figure 6A; Supplementary Excel 10). Precisely, there is a difference between the life stages of *I. sexdentatus* (ANOVA; df = 2, *p* < 0.01). However, the relative bacterial abundance between larval and pupal stages is marginally varied (contrast *t*-test; *p* = 0.099); adult bacterial assemblage was significantly different from larvae (contrast t-test; *p* < 0.01) but slightly diverse from pupae (contrast *t*-test; *p* = 0.054). The relative abundance of Bacteroidetes (phylum), *Enterobacteriaceae* (family)*, Pseudoxanthomonas, Pseudomonas, and Serratia* (genus) varied within the life stages of *I. sexdentatus* (Figures 6A,B,D–F). No abundance difference was observed for Firmicutes (Figure 6C).

### Wild adults vs. lab-bred adults

Similarly, the overall difference in bacterial abundance (eubacterial primers) between wild and breeding adult beetles was non-significant (ANOVA; df = 1, *p* = 0.076) (Supplementary Figure 6B; Supplementary Excel 10). However, the lab-bred *I. sexdentatus* adults showed a higher abundance of *Pseudomonas* and *Serratia* than wild adults (Figures 6J,L). Meanwhile, *Enterobacteriaceae* and *Pseudoxanthomonas* are more abundant in wild beetles (Figures 6H,K). No differences were observed in the relative abundance of Bacteroidetes and Firmicutes (Figures 6G,I).

#### *I. sexdentatus* vs. *I. acuminatus*

Comparing the two pine beetles, adults of *I. acuminatus* had significantly higher relative abundance than *I. sexdentatus* (ANOVA; df = 1, *p* < 0.001) (Supplementary Figure 6C; Supplementary Excel 10). The relative abundance of Bacteroidetes (phylum), *Enterobacteriaceae* (family), *Pseudomonas*, and *Pseudoxanthomonas* (genus) was significantly higher in *I. acuminatus* adults (Figures 6M,N,P,Q). However, *Serratia* (genus) abundance was greater in *I. sexdentatus* adults (Figure 6R). No significant differences in the relative abundance of Firmicutes were observed for any comparisons (Figures 6C,I,O).

## Discussion

Conifers have evolved a formidable defence against pests and pathogens (Klepzig et al., 1996; Krokene, 2015). In contrast, pests, including bark beetles and their associated microbiota, can successfully invade host trees by compromising host defence (Cheng et al., 2018). The insect microbiota is often influenced by the diet, sex, life stages, and the environment and facilitates the expansion of the ecological and evolutionary potential of their hosts (Yun et al., 2014; Lange et al., 2023). Recently, there has been growing interest in understanding the role of bark beetle-microbial association in shaping the plasticity of beetles. For instance, a recent study on Eurasian spruce bark beetles (ESBB), *I. typographus*, reported the developmental stage and the geo-location as drivers in shaping the beetle-microbial association (Chakraborty et al., 2023; Veselská et al., 2023; Baños-Quintana et al., 2024; Moussa et al., 2024). The gut bacterial dynamics in the pine beetle *Dendroctonus rhizophagus* revealed that the presence of persistent bacterial communities across the life stages of the beetle might be essential for ensuring certain physiological functions for the host (Briones-Roblero et al., 2017). However, information on the contribution of life stage and environment on the pine-feeding *Ips* bark beetles, *I. sexdentatus,* and *I. acuminatus* (Coleoptera: Curculionidae) is lacking. Hence, the present comparative study is focused on the influence of life stage and environment on the bacterial communities associated with the two pine-feeding *Ips* bark beetles. It is also important to mention that as our study focused on different life stages of the beetles, we did not separately evaluate sex-specific microbial assemblage in adult beetles. The sex-specific variation in the microbial association and their functional relevance will be an exciting avenue for future investigation. However, a recent gut proteomics study on ESBB failed to find any significant sex-specific protein expression difference in male and female adult beetles (Ashraf et al., 2023), suggesting conservation in gene expression. It will be interesting to see if such conservations exist in the case of microbial associations or not.

The bark beetle larvae spend their entire life gregariously feeding and developing under the bark. The larvae acquire microbial communities during feeding that mainly aid nutrient acquisition and detoxify plant defensive compounds (Peral-Aranega et al., 2020; Liu et al., 2022). During metamorphosis, beetles undergo complete structural changes from larvae to adults via the non-feeding pupal stage, which might lead to the gain or loss of certain microbiota (Sela et al., 2020). The compartmentalisation of the internal structures and organogenesis during metamorphosis leads to different physiological conditions, including redox potential, oxygen concentrations, and pH changes influencing the distribution and survival of the insect microbiota (Callegari et al., 2021). Recent findings by Peral-Aranega et al. (2023) revealed that the bacterial diversity in *I. typographus* reduced in the pupal stage compared to the larvae and regained in the adult stages, which was also observed with *I. acuminatus* beetles in our current study. However, there is no such trend in *I. sexdentatus* beetles. In *I. sexdentatus* beetles, bacterial richness and diversity gradually decreased after each life stage. Interestingly, adult beetles perform multiple duties on maturation, including feeding, host finding, and reproduction. Such responsibilities of the adult beetles can be associated with the maintenance and selectivity of their symbionts (Lemoine et al., 2020). Nevertheless, such possibilities need further experimental validation.

Our results demonstrated that the bacterial richness and diversity varied across the same developmental stages between the two *Ips* beetles. For instance, a high bacterial richness and diversity in *I. acuminatus* (IAC) adults was observed compared to *I. sexdentatus* (ISX). This interspecies variation in the bacterial community might be associated with their variable preference for pine trees. Although both *I. sexdentatus* and *I. acuminatus* feed on the same pine trees, they have particular preferences for pine trees. *I. acuminatus* beetles can attack healthy trees, whereas *I. sexdentatus* beetles are considered a secondary pest that prefers weakened and stressed pine trees to colonise (Wermelinger et al., 2008; Pineau et al., 2017a). In addition, *I. acuminatus* beetles infest young pine stands and plantations more aggressively than *I. sexdentatus* (Davydenko et al., 2017). Therefore, both *Ips* species might have differential resistance to host chemical defence that can influence bacterial partner selection and maintenance. Also, *I. acuminatus* beetles might require a wide range of bacterial species to deal with pine allelochemicals, which might explain the higher bacterial diversity in *I. acuminatus* adults compared to *I. sexdentatus* adults. In addition, *I. acuminatus* adult (wild) feeds on the upper canopy of the pine tree where the bark is thin, whereas *I. sexdentatus* adult (wild) beetles feed on the lower part of the bole where the bark is thick (Davydenko et al., 2017). Such habitat specialisation can be reflected by the differences in the bacterial diversity and richness between the two bark beetle species feeding on the same host. Recent research documented specific bark beetle fungal symbiotic system-mediated adaptation to feeding on different parts of the same conifer (spruce) hosts (Bai et al., 2024). Therefore, it can be assumed that microclimatic conditions can influence the symbiont association, richness and diversity in bark beetles. Nonetheless, such interpretations need to be further investigated in these beetles.

Moreover, for bark beetles and many other species, the competency of an organism to associate with other microorganisms can allow the species to thrive under challenging scenarios like climatic fluctuations and resources (Lemoine et al., 2020). Therefore, the fungus-feeding nature of *I. acuminatus* larvae might have some influence on its bacterial population dynamics. In addition, *I. acuminatus* also possess oral mycetangium for fungal transmission, which is rare to other *Ips* beetles, making them phylogenetically and morphologically distinct (Papek et al., 2024). The fungal species can be pathogenic to the host tree, while on the contrary, they may play nutritional roles in the *I. acuminatus* larvae (Villari et al., 2012) and might also interact with the bacterial communities residing in the developing larvae (Zhang et al., 2018). This could explain the lower bacterial richness in fungus-feeding *I. acuminatus* larvae but a higher bacterial diversity in *I. sexdentatus* larvae. However, dedicated investigations are needed to confirm the ecological relevance of such observations.

Laboratory adaptation and breeding conditions are essential factors that can affect bacterial associations unpredictably (Augustinos et al., 2019; Baños-Quintana et al., 2024). Compared to laboratory-bred beetles, broader and continuous environmental challenges in wild-collected beetles can encourage highly diversified and rich bacterial assemblage in wild-collected beetles. Furthermore, the bottleneck effect and high selective pressure are the key drivers in laboratory populations that often reduce symbiont load (Augustinos et al., 2019). Such observations may result in *I. sexdentatus* adults (wild) having higher bacterial diversity and richness than lab-bred adults (Table 2). However, no such trend was observed in *I. acuminatus*. Hence, dedicated investigations are needed to understand the ecological relevance of such observation. It is worth mentioning that the beetles were collected in different years; hence, the observed variation can also be due to feeding on different wood under different environmental conditions. A comparison of lab-bred (control wood, fed wood) and wild-collected (*I. sexdentatus* control and fed wood) wood samples for the same species (*I. sexdentatus*) has revealed that each wood sample poses a distinct bacterial population (Supplementary Excel 9) endorsing our prediction.

Although both pine beetles have distinct bacterial communities influenced by microclimatic conditions, canopy preference, metamorphosis, and feeding behaviour, our study revealed a core bacteriome across all developmental stages of two pine beetles (Figure 2C). Nevertheless, the bacterial composition did not vary, but there were distinct variations in the relative abundance of the bacterial genera. The core bacterial communities within the developmental stages of the two pine-feeding beetles were dominated by bacterial genera belonging to *Erwiniaceae* and *Pseudomonaceae.* A similar observation was reported in other beetles (Cambronero-Heinrichs et al., 2023; Peral-Aranega et al., 2023; Veselská et al., 2023). Our results documented the prevalence of *Pseudomonas* in the pine-feeding larvae. *Pseudomonas* is one of the most prominent bacterial genera isolated from different bark beetle species and across life stages (Saati-Santamaría et al., 2021), suggesting their pivotal role in the survival of the beetles. For example, the bacterial strains *P. bohemica* and *P. typographi* that were consistently present in *I. typographus* are reported to produce lytic enzymes and antifungal compounds (Peral-Aranega et al., 2023). Other abundant bacterial genera, including *Serratia*, *Pseudoxanthomonas, Sphingmonas, Acinetobacter* were present throughout the developmental stages of the two beetles, *I. acuminatus* and *I. sexdentatus.* The persistence of such bacterial strains across the life stages indicates their pivotal role in bark beetle holobiont (Cardoza et al., 2009; Fang et al., 2020; Ge et al., 2021; Peral-Aranega et al., 2023). Different strains of *Pseudomonas*, *Serratia*, *Pseudoxanthomonas* have been shown to be associated with lignocellulose degradation (Kumar et al., 2015; García-Fraile, 2018; Saati-Santamaría et al., 2021), indicating their role in the provision of nutrients to the beetles. For instance, *Pseudomonas putida* and *P. azotoformans* isolated from *Dendroctonus rhizophagus* were shown to have cellulolytic, amylolytic, xylanolytic, lipolytic, and esterase activity (Briones-Roblero et al., 2017). Our findings revealed a high abundance of *Serratia* in adults and the pupal stage of the pine beetles. Such observation was corroborated by Fang et al. (2020) where *Serratia* was dominant in adults and pupal stages of *Ips typographus* (Fang et al., 2020). Bacterial strains *Serratia marcescens*, and *Pseudomonas mandelii*, isolated from *Dendroctonus ponderosae* adults, have been shown to degrade plant defense chemicals (monoterpenes) (Boone et al., 2013). Additionally, bacterial strains from the core consortium, including *Pseudomonas*, *Acinetobacter*, and members of the *Erwiniaceae* family in the two pine beetle species, were reported to produce siderophores, aiding in competition by limiting the availability of iron in the environment (Peral-Aranega et al., 2023). Such results can provide some working hypothesis to follow up in future with individual stains isolated from these beetles via culture dependent methods.

Our study also reveals beetle-mediated alterations in wood bacterial assemblage, similar to insect herbivores that reshape the native plant leaf microbiome (Humphrey and Whiteman, 2020). However, the degree of overlap or distinctiveness between insect and host (wood) microbiome remains ambiguous (Pirttilä et al., 2023). Precisely, higher sharing between fed wood and beetle samples followed by lower bacterial diversity in fed wood samples than in control wood can be due to several reasons. It can be presumed that bacterial association in both beetle species is influenced by the horizontal transfer of bacteria from the pine host. Further studies are required to comprehend the ecological relevance of beetle-mediated tree bacteriome alteration.

### Limitation

The sample size in our study is relatively small, with fewer individuals per replicate, which may have reduced our ability to eliminate transient species while characterising the core microbiome. Secondly, we collected samples from one forest location in the Czech Republic, and that geographical distribution might introduce a potential confounder that could influence the composition of the pine beetle bacteriome, leading to some biases in the results. Moreover, 16S rRNA amplicon sequencing restricted our taxonomic identification to the genus level, potentially missing crucial functional associations at the species or strain levels. Nonetheless, our study offers valuable hypotheses for delving into pine bark beetle symbiosis in future.

## Conclusion

Our comparative metagenomic study reported the impact of life stage and lab-breeding on the bacterial communities in two pine-feeding beetles. The core bacterial genera, including *Pseudomonas*, *Pseudoxanthomonas,* and *Acinetobacter* were dominant in the pine beetles. The bacterial diversity and richness varied significantly across life stages, wild collected, and laboratory populations. *I. sexdentatus* larvae represented significantly higher bacterial diversity and community richness and evenness compared to other developmental stages, while *I. acuminatus* adults demonstrated higher bacterial richness with no significant variation in the bacterial diversity and richness between the life stages. Lab-bred and wild beetles showed distinct bacterial diversity and richness. Beetle feeding substantially influenced the host bacteriome at the feeding galleries. Further downstream studies to characterise the key highly abundant and transient bacterial species will provide new ecological insights into pine bark beetle symbiosis, particularly concerning their metabolic capacities, interactions with other symbionts, and roles in the detoxification of conifer allelochemicals. Essential bacterial partners can also be used for microbiome-based sustainable pest management practises as carriers for dsRNA against bark beetles (Whitten et al., 2016; Qadri et al., 2020; Joga et al., 2021; Gupta et al., 2023).

## Data Availability

The bacteriome dataset in the study is available under NCBI Bio-project PRJNA854390.

## Footnotes

^1^http://ccb.jhu.edu/software/FLASH/
