# Supplementary material for: Comparative metagenomic study unveils new insights on bacterial communities in two pine-feeding Ips beetles (Coleoptera: Curculionidae: Scolytinae)
Source: Front Microbiol. 2024 Oct 9;15:1400894. doi: 10.3389/fmicb.2024.1400894 (PMC11496174; doi:10.3389/fmicb.2024.1400894)

Supplementary figure 1

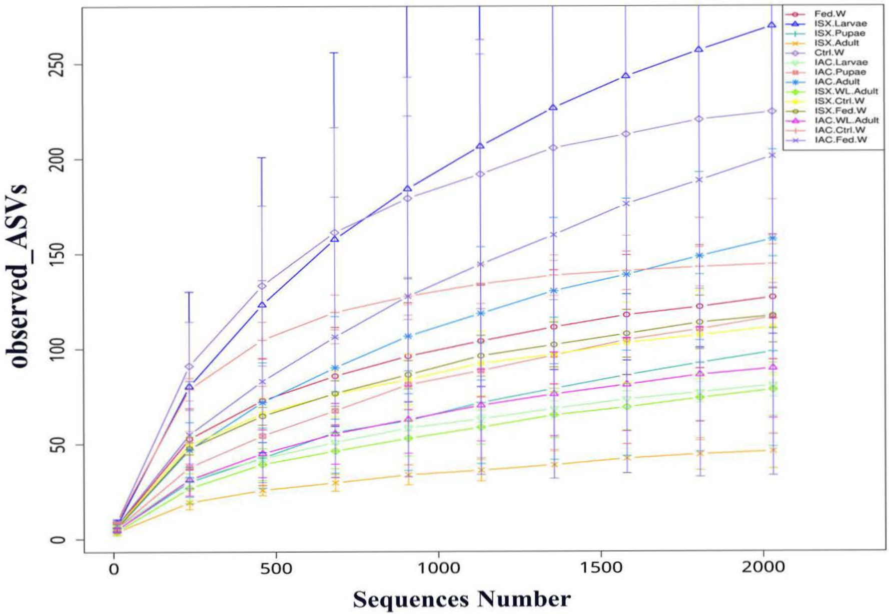

Supplementray Figure 2

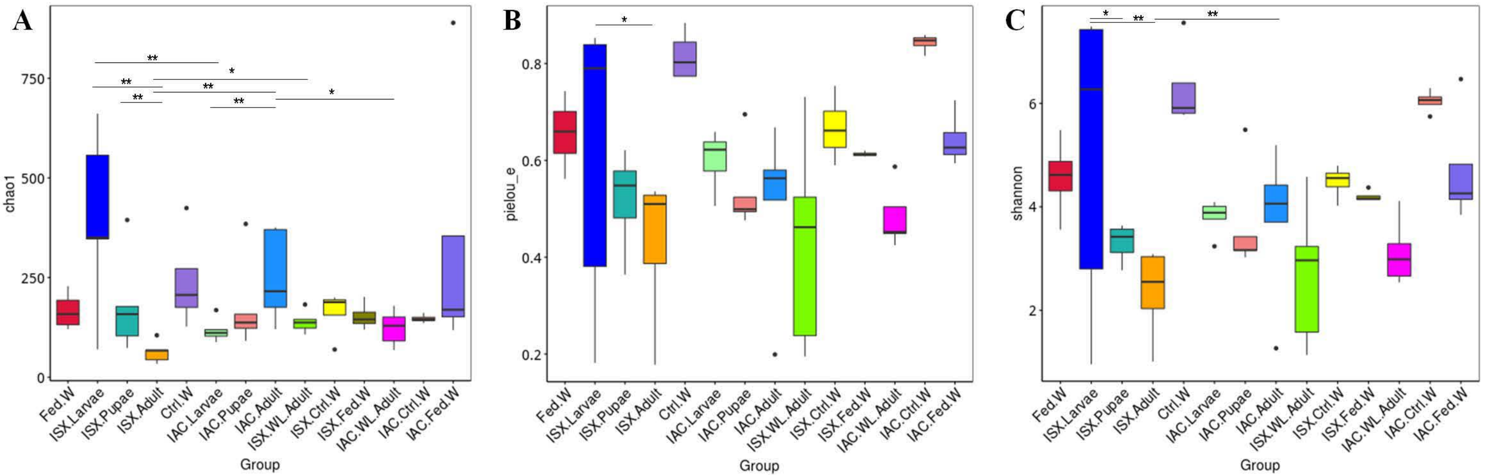

Supplementary figure 3

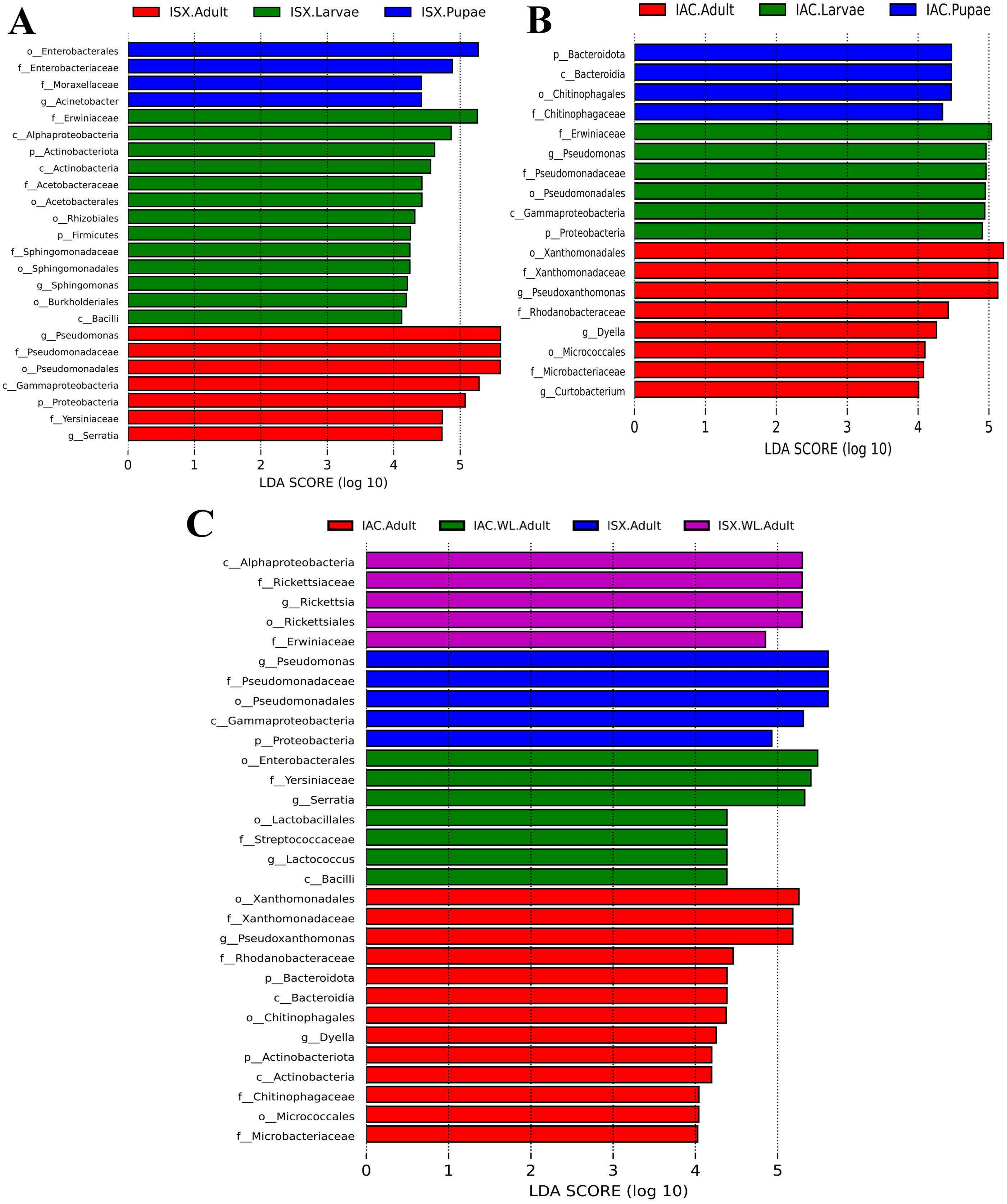

Supplementary figure 4

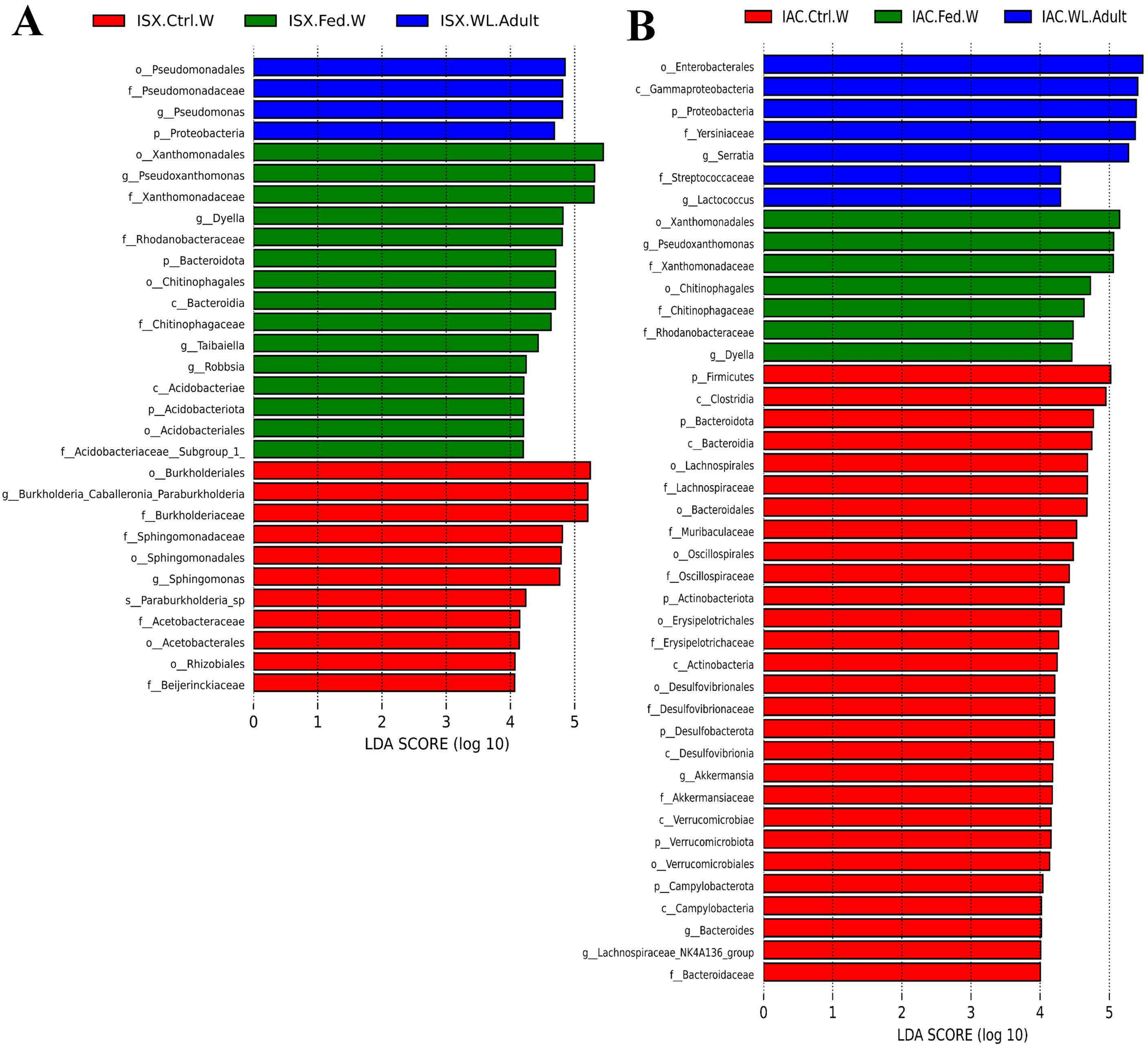

Supplementary Figure 5

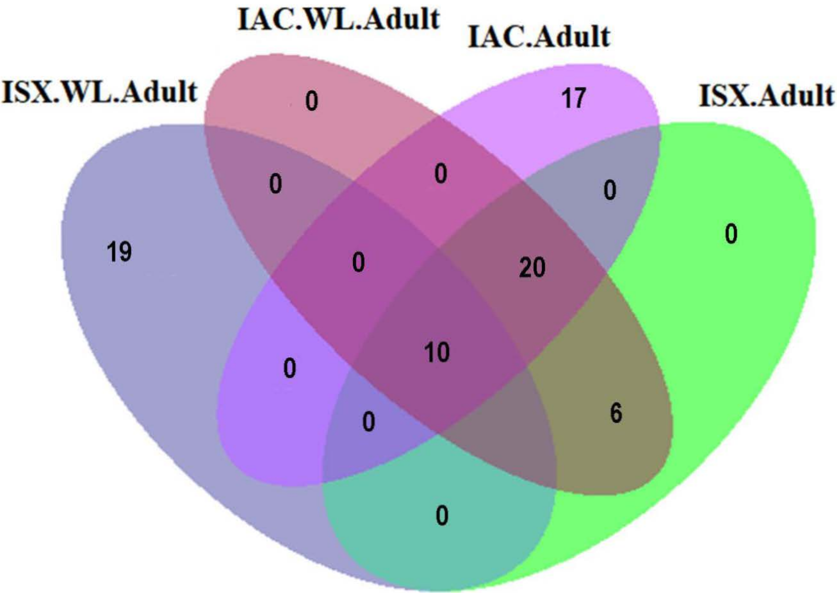

Supplementary Figure 6

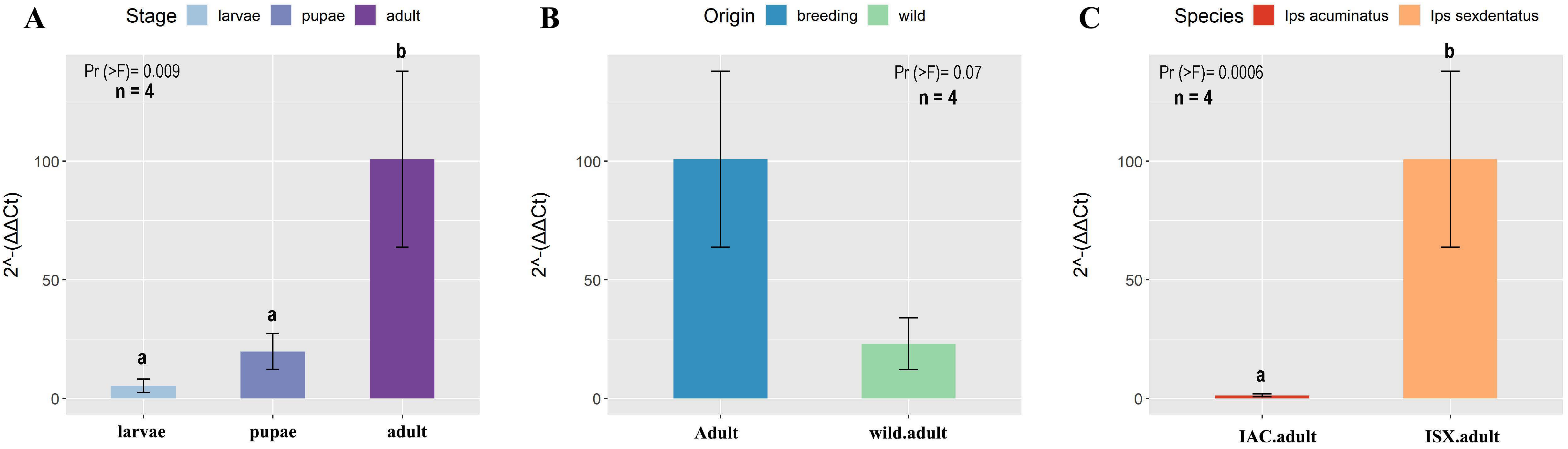

Supplement: Supplementary file 1 [file Data_Sheet_1.pdf]
