# Supplementary material for: Comparative metagenomic study unveils new insights on bacterial communities in two pine-feeding Ips beetles (Coleoptera: Curculionidae: Scolytinae)
Source: Front Microbiol. 2024 Oct 9;15:1400894. doi: 10.3389/fmicb.2024.1400894 (PMC11496174; doi:10.3389/fmicb.2024.1400894)
Supplement: Supplementary file 2 [file Data_Sheet_2.pdf]

Supplementary Tables

Supplementary Table 1| **ADONIS analysis evaluating the significant difference between different life stages of both *Ips* pine beetles** (ISX-*I. sexdentatus*, IAC-*I. acuminatus*) a**nd wood samples** (Df = degree of freedom, SS = sums of squares of deviations, MS = SS/Df, F. Model = F-test value, R2 = the ratio of grouping variance and total variance). Values in parentheses illustrate Residual Error. The p-value represents the significant variation between different stages of both bark beetles.

Group	Df	SS	MS	F.Model	R2	Pr(>F)
ISX.Larvae-ISX.Pupae	1(8)	1.16048 (2.01928)	1.16048 (0.25241)	4.59759	0.36496 (0.63504)	0.009
ISX.Larvae-ISX.Adult	1(8)	1.24029 (2.19883)	1.24029 (0.27485)	4.51253	0.36064 (0.63936)	0.007
ISX.Larvae-IAC.Larvae	1(8)	1.5418 (1.59931)	1.5418 (0.19991)	7.71232	0.49085 (0.50915)	0.007
ISX.Pupae-ISX.Adult	1(8)	1.01752 (1.93704)	1.01752 (0.24213)	4.20236	0.34439 (0.65561)	0.019
ISX.Pupae-IAC.Pupae	1(8)	1.31166 (1.72179)	1.31166 (0.21522)	6.0944	0.4324 (0.5676)	0.007
ISX.Adult-IAC.Adult	1(8)	1.42459 (1.74022)	1.42459 (0.21753)	6.54903	0.4324 (0.5676)	0.006
ISX.Adult-ISX.WL.Adult	1(8)	1.02731 (2.19077)	1.02731 (0.27385)	3.7514	0.31923 (0.68077)	0.008
IAC.Larvae-IAC.Pupae	1(8)	0.61515 (1.30182)	0.61515 (0.16273)	3.78026	0.3209 (0.6791)	0.01
IAC.Larvae-IAC.Adult	1(8)	0.86162 (1.1407)	0.86162 (0.14259)	6.04274	0.43031 (0.56969)	0.013
IAC.Pupae-IAC.Adult	1(8)	0.50118 (1.52498)	0.50118 (0.19062)	2.62918	0.24736 (0.75264)	0.017

IAC.Adult-IAC.WL.Adult	1(8)	1.05213 (1.61296)	1.05213 (0.20162)	5.21837	0.39478 (0.60522)	0.008
ISX.WL.Adult-ISX.Ctrl.W	1(7)	0.89651 (1.61516)	0.89651 (0.23074)	3.88543	0.35694 (0.64306)	0.009
ISX.WL.Adult-ISX.Fed.W	1(7)	1.03372 (1.14315)	1.03372 (0.16331)	6.32989	0.47486 (0.52514)	0.006
ISX.WL.Adult-IAC.WL.Adult	1(8)	0.80898 (2.06351)	0.80898 (0.25794)	3.13633	0.28163 (0.71837)	0.017
ISX.Ctrl.W-ISX.Fed.W	1(6)	0.89886 (0.49335)	0.89886 (0.08223)	10.93171	0.64564 (0.35436)	0.022
IAC.WL.Adult-IAC.Ctrl.W	1(7)	1.03796 (1.33903)	1.03796 (0.19129)	5.42614	0.43667 (0.56333)	0.012
IAC.WL.Adult-IAC.Fed.W	1(7)	0.86866 (1.36594)	0.86866 (0.19513)	4.45164	0.38873 (0.61127)	0.006

Supplementary Table 2| **ANOSIM analysis representing the magnitude of variation between different life stages of both *Ips* pine beetles** (ISX-*I. sexdentatus*, IAC-*I. acuminatus*). In ANOSIM analysis, the positive R values indicate significant differences between the bacterial communities present at different life stages of both beetles. P-value < 0.05 indicates significant differences between different stages of both bark beetles and wood samples.

Group	R-value	P-value
ISX.Larvae-ISX.Pupae	0.68	0.017
ISX.Larvae-ISX.Adult	1	0.006
ISX.Larvae-IAC.Larvae	1	0.006
ISX.Pupae-ISX.Adult	0.548	0.013
ISX.Pupae-IAC.Pupae	0.736	0.009
ISX.Adult-IAC.Adult	1	0.007
ISX.Adult-ISX.WL.Adult	0.852	0.006
IAC.Larvae-IAC.Pupae	0.548	0.009
IAC.Larvae-IAC.Adult	0.748	0.009
IAC.Pupae-IAC.Adult	0.368	0.019
IAC.Adult-IAC.WL.Adult	0.828	0.008
ISX.WL.Adult-ISX.Ctrl.W	0.68125	0.008
ISX.WL.Adult-ISX.Fed.W	0.56562	0.023
ISX.WL.Adult-IAC.WL.Adult	0.682	0.007
ISX.Ctrl.W-ISX.Fed.W	0.85417	0.025
IAC.WL.Adult-IAC.Ctrl.W	0.89375	0.005
IAC.WL.Adult-IAC.Fed.W	0.71875	0.01

Supplementary Table 3| Relative abundance of the biomarkers obtained from LEfSe analysis

Groups	Biomarker relative abundance (%)		
ISX.Larvae vs ISX.Pupae vs ISX.Adult	**ISX.Larvae**	**ISX.Pupae**	**ISX.Adult**
	Actinobacteria (8%)<br>Bacilli (3%)<br>Alphaproteobacteria (15%)<br>Acetobacterales (5%)<br>Sphingomonadales (4%)<br>Burkholderiales (3%)<br>Rhizobiales (4%)<br>*Sphingomodaceae* (3%)<br>*Erwiniaceae* (36%)<br>*Acetobacteraceae* (5%)	Enterobacterales (54%)<br>*Moraxellaceae* (5%)<br>*Enterobacteriaceae* (15%)	Gamaproteobacteria (99%)<br>Pseudomonadales (82%)<br>*Yersiniaceae* (10%)<br>*Pseudomonadaceae* (82%)
IAC.Larvae vs IAC.Pupae vs IAC.Adult	**IAC.Larvae**	**IAC.Pupae**	**IAC.Adult**
	Gammaproteobacteria (95%)<br>Pseudomonadales (20%)<br>*Erwiniaceae* (22%)<br>*Pseudomonadaceae* (20%)	Bacteroidia (7%),<br>Chitinophagales (7%)<br>*Chitinophagaceae* (5%)	Micrococcales (2%)<br>Xanthomonadales (35%)<br>*Rhodanobactericeae* (5%)<br>*Xanthomonadeceae* (30%)<br>*Microbactericeae* (2%)
ISX.WL.Adult vs ISX.Ctrl.W vs ISX.Fed.W	**ISX.WL.Adult**	**ISX.Ctrl.W**	**ISX.Fed.W**
	Pseudomonadaceae (12%)<br>Pseudomonadales (13%)	Acidobacteriae (3%)<br>Burkholderiales (32%)<br>Acidobacteriales (2%)<br>Sphingomonadales (12%)<br>Rhizobiales (2%)<br>*Burkholderiaceae* (32%)<br>*Beijerinckiaceae* (2%)<br>*Sphingomodadaeceae* (12%)	Bacterodiota (10%),<br>Acidobacteriae (3%)<br>Acidobacteriales (3%)<br>Chitinophagales (10%)<br>Xanthomonadales (54%)<br>*Acidobacteriaceae* _(Subgroup_1) (3%),<br>*Chitinophagaceae* (8%),<br>*Rhodanobacteraceae* (13%)<br>*Xanthomonadaceae* (40%)
IAC.WL.Adult vs IAC.Ctrl.W vs IAC.Fed.W	**IAC.WL.Adult**	**IAC.Ctrl.W**	**IAC.Fed.W**
	Gammaproteobacteria (88%)<br>Enterobacterales (78%)<br>*Streptococcaceae* (4%)<br>*Yersiniaceae* (50%)	Actinobacteria (1%)<br>Bacteroidia (14%)<br>Verrucomicrobiae (3%)<br>Lachnospirales (9%)<br>Erysipelotrichales (4%)<br>Bacteroidales (9%)<br>Desulfovibrionales (3%)<br>Verrucomicrobiales (3%)<br>Oscillospirales (6%)<br>*Bacterodiaceae* (1%)<br>*Desulfovibrionaceae* (3%),<br>*Akkermansiaceae* (2%)<br>*Muribaculaceae* (6%)<br>*Campylobacteria* (2%)<br>*Desulfovibrionia* (3%)<br>*Erysipelotrichaceae* (4%)<br>*Lachnospiraceae* (9%)<br>*Oscillospiraceae* (5%)<br>*Clostridia* (16%)	Chitinophagales (14%)<br>Xanthomonadales (36%)<br>*Chitinophagaceae* (10%)<br>*Rhodanobacteraceae* (7%)<br>*Xanthomonadaceae* (28%)

Supplementary Table 4| Relative abundance of the biomarkers (obtained from LEfSe analysis) present in wild and lab-bred adults of two pine-feeding beetles. IAC. Adult- *I. acuminatus* lab-bred adult, IAC. WL.adult- *I. acuminatus* wild-collected adults, ISX. Adult- *Ips sexdentatus* lab-bred adult, ISX. WL.adult- *I. sexdentatus* wild-collected adults.

**Groups**	**Biomarker relative abundance (%)**	
	**IAC.Adult**	**IAC.WL.Adult**
IAC.Adult vs IAC.WL.Adult	Actinobacteria (3%),<br>Bacteroidia (5%),<br>Micrococcales (2%),<br>Chitinophagales (5%),<br>Xanthomonadales (35%)<br>*Rhodanobactericeae* (5%)<br>*Xanthomonadeceae* (30%)<br>*Microbacteriaceae* (2%)<br>*Chitinophagaceae* (2%)	Bacilli (5%)<br>*Yersiniaceae* (50%),<br>Enterobacterales (78%)<br>*Streptococcaceae* (4%)<br>*Lactobacillales* (4%)
	**ISX.Adult**	**ISX.WL.Adult**
ISX.Adult vs ISX.WL.Adult	Gammaproteobacteria (99%)<br>*Pseudomonadales* (82%)<br>*Pseudomonadaceae* (82%)	Alphaproteobacteria (34%),<br>Rickettsiales (34%)<br>*Rickettsiaceae* (34%)<br>*Erwiniaceae* (17%)

Supplementary Table 5| **Selected bacterial primers used for the real-time quantitative PCR assay.**

Primer Code	Primer name	Primer Sequences (5’-3’)	Amplicon length (bp)	Annealing temperature (℃)	Reference
Eub	Eubacterial primer	Eub338F: ACTCCTACGGGAGGCAGCAG<br>Eub518R: ATTACCGCGGCTGCTGG	180	60	(1)
Cfb	Bacteroidetes	Cfb319: GTACTGAGACACGGACCA<br>Eub518R: ATTACCGCGGCTGCTGG	220	60	(2)
Lgc	Firmicutes	Lgc353: GCAGTAGGGAATCTTCCG<br>Eub518R: ATTACCGCGGCTGCTGG	180	60	
Eco	*Enterobacteriaceae*	Eco1457F: CATTGACGTTACCCGCAGAAGAAGC<br>Eco1652R: CTCTACGAGACTCAAGCTTGC	195	60	(3)
Pse	*Pseudomonas*	Pseu-F: ACCGCATACGTCCTACGG<br>Pseu-R: CGAAGACCTTCTTCACACACG	251	60	(4)
Psx	*Pseudoxanthomoas*	PsxF1: TGGATGTTGGGTTCAACTTGG<br>PsxR1: GACCAGGTAAGGTTCTTCGC	165	60	This study
Ser	*Serratia*	SerF1: TAGCACAGGAGAGCTTGCTCT<br>SerR1: CGAAGGTCCCCCACTTTGGTC	142	60	This study

References:

1. Bergmark, L., Poulsen, P.H.B., Al-Soud, W.A., Norman, A., Hansen, L.H. and Sørensen, S.J., 2012. Assessment of the specificity of Burkholderia and Pseudomonas qPCR assays for detection of these genera in soil using 454 pyrosequencing. *FEMS microbiology letters*, *333*(1), pp.77-84.
2. Fierer, N., Jackson, J.A., Vilgalys, R. and Jackson, R.B., 2005. Assessment of soil microbial community structure by use of taxon-specific quantitative PCR assays. *Applied and environmental microbiology*, *71*(7), pp.4117-4120.
3. Bartosch, S., Fite, A., Macfarlane, G.T. and McMurdo, M.E., 2004. Characterization of bacterial communities in feces from healthy elderly volunteers and hospitalized elderly patients by using real-time PCR and effects of antibiotic treatment on the fecal microbiota. *Applied and environmental microbiology*, *70*(6), pp.3575-3581.
4. Najafpour, B., Pinto, P.I., Canario, A.V.M. and Power, D.M., 2022. Quantifying dominant bacterial genera detected in metagenomic data from fish eggs and larvae using genus-specific primers. *MicrobiologyOpen*, *11*(3), p.e1274.
